# Information-Restricted Neural Language Models Reveal Different Brain Regions’ Sensitivity to Semantics, Syntax, and Context

**DOI:** 10.1162/nol_a_00125

**Published:** 2023-12-14

**Authors:** Alexandre Pasquiou, Yair Lakretz, Bertrand Thirion, Christophe Pallier

**Affiliations:** Cognitive Neuroimaging Unit (UNICOG), NeuroSpin, National Institute of Health and Medical Research (Inserm) and French Alternative Energies and Atomic Energy Commission (CEA), Frédéric Joliot Life Sciences Institute, Paris-Saclay University, Gif-sur-Yvette, France; Models and Inference for Neuroimaging Data (MIND), NeuroSpin, French Alternative Energies and Atomic Energy Commission (CEA), Inria Saclay, Frédéric Joliot Life Sciences Institute, Paris-Saclay University, Gif-sur-Yvette, France

**Keywords:** context, encoding models, fMRI, LLM, semantics, syntax

## Abstract

A fundamental question in neurolinguistics concerns the brain regions involved in syntactic and semantic processing during speech comprehension, both at the lexical (word processing) and supra-lexical levels (sentence and discourse processing). To what extent are these regions separated or intertwined? To address this question, we introduce a novel approach exploiting neural language models to generate high-dimensional feature sets that separately encode semantic and syntactic information. More precisely, we train a lexical language model, GloVe, and a supra-lexical language model, GPT-2, on a text corpus from which we selectively removed either syntactic or semantic information. We then assess to what extent the features derived from these information-restricted models are still able to predict the fMRI time courses of humans listening to naturalistic text. Furthermore, to determine the windows of integration of brain regions involved in supra-lexical processing, we manipulate the size of contextual information provided to GPT-2. The analyses show that, while most brain regions involved in language comprehension are sensitive to both syntactic and semantic features, the relative magnitudes of these effects vary across these regions. Moreover, regions that are best fitted by semantic or syntactic features are more spatially dissociated in the left hemisphere than in the right one, and the right hemisphere shows sensitivity to longer contexts than the left. The novelty of our approach lies in the ability to control for the information encoded in the models’ embeddings by manipulating the training set. These “information-restricted” models complement previous studies that used language models to probe the neural bases of language, and shed new light on its spatial organization.

## INTRODUCTION

Understanding the neural bases of language processing has been one of the main research efforts in the neuroimaging community for the past decades (see, e.g., Binder et al., 2009; Friederici, 2011, for reviews). However, the complex nature of language makes it difficult to discern how the various processes underlying language processing are topographically and dynamically organized in the human brain, and therefore many questions remain open to this date.

One central open question is whether semantic and syntactic information are encoded and processed jointly or separately in the human brain. Language comprehension requires to access word meanings (lexical semantics), but also to compose these meanings to construct the meaning of entire sentences. In languages such as English, the meaning of a sentence strongly depends on word order—for example, “The boy kissed the girl” has a different meaning than “The girl kissed the boy” although both sentences contain the exact same words. (In other languages, inflectional cues rather than word order signal the roles of words in the sentence.) Importantly, meaning construction of new sentences would be roughly done in the same way if only the structure of the sentences remains the same (“The X kissed the Y”), independently of the lexical meanings of the single nouns in the sentences (“boy” and “girl”). This combinatorial property of language allows us to construct meanings of sentences that we have never heard before and suggests that it might be computationally advantageous for the brain to have developed neural mechanisms for composition that are separate from those dedicated to the processing of lexico-semantic content. Such neural mechanisms for composition would be sensitive to only the abstract structure of sentences and would implement the syntactic rules according to which sentence parts should be composed.

Following related considerations, the dominant view over the past decades claimed that syntactic information is represented and processed in specialized brain regions, akin to the classic modular view (Chomsky, 1984; Fodor, 1983). Neuronal modularity of language processing gained support from early lesion studies suggesting that syntactic processing takes place in localized and specialized brain regions such as Broca’s area, showing double dissociations between syntactic and semantic processing (Caramazza & Zurif, 1976; Goodglass, 1993). Neuroimaging studies (Embick, 2000; Friederici et al., 2006; Friederici et al., 2017; Garrard et al., 2004; Grodzinsky & Santi, 2008; Hagoort, 2014; Hashimoto & Sakai, 2002; Matchin & Hickok, 2020; Pallier et al., 2011; Shetreet & Friedmann, 2014; Vigliocco, 2000) as well as simulation work on language acquisition and processing in artificial neural language models (Lakretz et al., 2019; Lakretz et al., 2021; O’Reilly & Frank, 2006; Russin et al., 2019; Ullman, 2004) have provided further support to this view since then.

However, in parallel, an opposing view has argued that semantics and syntax are processed in a common distributed language processing system (Bates & Dick, 2002; Bates & MacWhinney, 1989; Dick et al., 2001). Recent work in support of this view has raised concerns regarding the replicability of some of the early results from the modular view (Siegelman et al., 2019) and provided evidence that semantic and syntactic processing in the language network might not be so easily dissociated from one another (Fedorenko et al., 2020; Mollica et al., 2020).

Neuroimaging studies, cited to defend one or the other view, have mainly relied on one of two methodological approaches: on the one hand, controlled experimental paradigms, which manipulate the words or sentences (Bottini et al., 1994; Caplan et al., 1998; Mazoyer et al., 1993; Pallier et al., 2011; Stromswold et al., 1996) and, on the other hand, naturalistic paradigms that make use of stimuli closer to what one could find in a daily environment. The former approach probes linguistic dimensions in one of the following ways: varying the presence or absence of syntactic or semantic information (Friederici et al., 2003; Friederici et al., 2010) or varying the syntactic structure difficulty or the semantic interpretation difficulty (e.g., Cooke et al., 2001; Friederici et al., 2009; Kinno et al., 2008; Newman et al., 2010; Santi & Grodzinsky, 2010). However, the conclusions from such studies may be bounded to the peculiarity of the task and setup used in the experiment (Nastase et al., 2020). To overcome these shortcomings, over the last years, researchers have become increasingly interested in data using *ecological* paradigms in which participants are engaged in more natural tasks, such as conversation or story listening (LeBel et al., 2023; Lerner et al., 2011; Nastase et al., 2021; Pasquiou et al., 2022; Regev et al., 2013; Wehbe et al., 2014). This avoids any task-induced bias and takes into consideration both lexical and supra-lexical levels of syntax and semantic processing. Integrating supra-lexical level information is essential for understanding language processing in the brain, because the lexical-semantic information of a word and the resulting semantic compositions depend on its context.

More recently, following advances in natural language processing (NLP), neural language models have been increasingly employed in the analysis of data collected from ecological paradigms. Neural language models are models based on neural networks, which are trained to capture joint probability distributions of words in sentences using next-word, or masked-word prediction tasks (e.g., Devlin et al., 2019; Elman, 1991; Pennington et al., 2014; Radford et al., 2019). By doing so, the models have to learn semantic and syntactic relations among word tokens in the language. To study brain data collected from ecological paradigms, neural language models are presented with the same text stimuli, then their activations (aka embeddings) are extracted and used to fit and predict the brain data (Caucheteux & King, 2022; Huth et al., 2016; Pasquiou et al., 2022; Wehbe et al., 2014). This approach has led to several discoveries, such as wide networks associated with semantic processing uncovered by Huth et al. (2016) using word embeddings (see also Pereira et al., 2018), or context-sensitivity maps discovered by Jain and Huth (2018) and Toneva and Wehbe (2019).

Despite these advances and extensive neuroscientific and cognitive explorations, the neural bases of semantics, syntax and the integration of contextual information still remain debated. In particular, a central puzzle remains in the field: Some studies investigating syntax and semantics found vastly distributed networks when using naturalistic stimuli (Caucheteux et al., 2021; Fedorenko et al., 2020), and others found more localized activations for syntax, typically in inferior frontal and posterior temporal regions, when using constrained experimental paradigms (e.g., Matchin et al., 2017; Pallier et al., 2011). Thus, whether there is a hierarchy of brain regions integrating contextual information or the extent to which syntactic information is independently processed from semantic information, in at least some brain regions, remains largely debated to date.

So far, insights from neural language models about this central puzzle were also rather limited. This is mostly due to the complexity of the models in terms of size, training and architecture. This complexity makes it difficult to identify how and what information is encoded in their latent representations, and how to use their embeddings to study brain function.

Caucheteux et al. (2021) used a neural language model, GPT-2, in an novel way to separate semantic and syntactic processing in the brain. Specifically, using a pre-trained GPT-2 model, they built syntactic predictors by averaging the embeddings of words from sentences that shared syntactic but no semantic properties and used them to identify syntactic-sensitive brain regions. They defined as semantic-sensitive brain regions, the regions that were better predicted by the GPT-2’s embeddings computed on the original text, compared to the syntactic predictors. They observed that syntax and semantics, defined in this way, rely on a common set of distributed brain areas.

Jain and Huth (2018) used pre-trained long short-term memory (LSTM) models to study context integration. They varied the amount of context used to generate word embeddings, and obtained a map indicating brain regions’ sensitivity to different sizes of context.

### The Current Study

We propose a new approach to tackle the questions of syntactic versus semantic processing and contextual integration, by fitting brain activity with word embeddings derived from *information-restricted* models. By this, we mean that the models are trained on text corpora from which specific types of information (syntactic, semantic, or contextual) were removed. We then assess the ability of these information-restricted models to fit brain activations, and compare it to the predictive performance of a neural model trained on the integral dataset.

**Figure 1.**
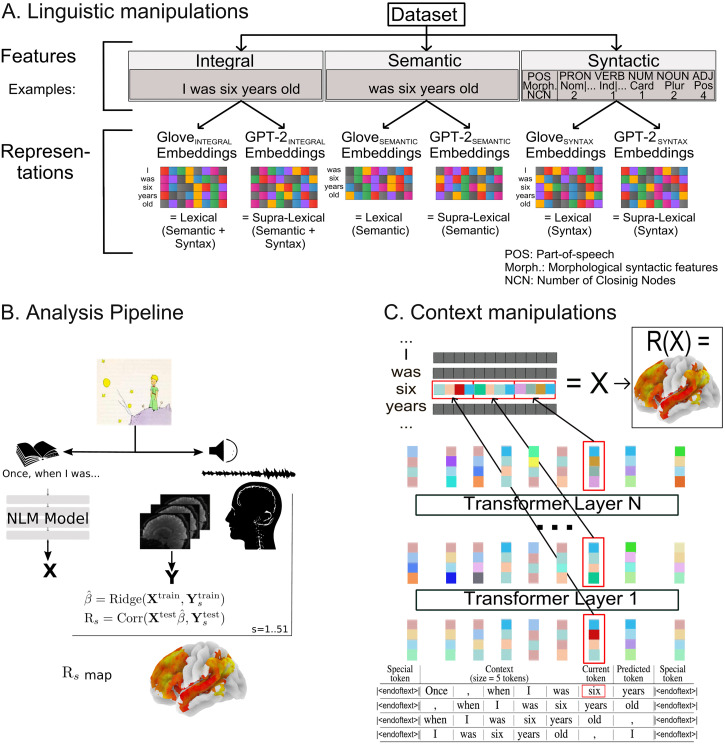
Experimental setup. (A) A corpus of novels was used to create a dataset from which we extracted three different sets of features: (i) integral features, comprising all tokens (words+punctuation); (ii) semantic features, comprising only the content words; (iii) syntactic features, comprising syntactic characteristics (part-of-speech, morphological syntactic characteristics, and number of closing nodes) of all tokens. GloVe and GPT-2 models were trained on each feature space. (B) fMRI scans of human participants listening to an audiobook were obtained. The associated text transcription was input to neural models, yielding embeddings that were convolved with an haemodyamic kernel and fitted to brain activity using a Ridge regression. Brain maps of cross-validated correlation between encoding models’ predictions and fMRI time series were computed. (C) To study sensitivity to context, a GPT-2 model was trained and tested on input sequences of bounded context length (5, 15, and 45). The resulting representations were then used to predict fMRI activity.

More precisely, we created a text corpus of novels from the Gutenberg Project (https://www.gutenberg.org) and used it to define three different sets of features: (i) *integral features*, the full text from the corpus; (ii) *semantic features*, the content words from the corpus; and (iii) *syntactic features*, where each word and punctuation sign from the corpus is replaced by syntactic characteristics. We then trained two types of models on each feature space: a noncontextual model, GloVe (Pennington et al., 2014), and a contextual model, GPT-2 (Radford et al., 2019; see Figure 1A). The text transcription of the audiobook, which participants listened to in the scanner, was then presented to the neural language models from which we derived embedding vectors. After fitting these embedded representations to functional magnetic resonance imaging (fMRI) brain data with linear encoding models, we computed the cross-validated correlations between the encoding models’ predicted time courses and the observed time series. In a first set of analyses, this allowed us to quantify the sensitivity to syntactic and semantic information in each voxel (Figure 1B). In a second set of analyses, we identified brain regions integrating information beyond the lexical level. We first compared the contextual model (GPT-2) and the noncontextual model (GloVe), before investigating the brain regions processing short (5 words), medium (15 words) and long (45 words) contexts, using a noncontextualized GloVe model as a 0-context baseline (see Figure 1C).

## METHODS AND MATERIALS

### Creation of Datasets: Semantic, Syntactic and Integral Features

We selected a collection of English novels from Project Gutenberg (www.gutenberg.org; data retrieved on February 21, 2016). This *integral dataset* comprised 4.4GB of text for training purposes and 1.1GB for validation. From it, we created two information-restricted datasets: the *semantic dataset* and the *syntactic dataset*. In the *semantic dataset*, only content words were kept, while all grammatical words and punctuation signs were filtered out. In the *syntactic dataset*, each token (word or punctuation sign) was replaced by an identifier encoding a triplet (POS, Morph, NCN), where POS is the part-of-speech computed using Spacy (Honnibal & Montani, 2017), Morph corresponds to the morphological features obtained from Spacy, and NCN stands for the number of closing nodes in the parse tree, at the current token, computed using the Berkeley Neural Parser (Kitaev & Klein, 2018) available with Spacy.

In this article, we refer to the content of the integral dataset as integral features, the content of the semantic dataset as semantic features, and the content of the syntactic dataset as syntactic features. Examples of integral, semantic, and syntactic features are given in Appendix A in the Supporting Information.

### GloVe Training

GloVe (Global Vectors for Word Representation) relies on the co-occurence matrix of words in a given corpus to generate fixed embedding vectors that capture the distributional properties of the words (Pennington et al., 2014). Using the open-source code provided by Pennington et al. (https://nlp.stanford.edu/projects/glove/), we trained GloVe on the three datasets (integral, semantic, and syntactic), setting the context window size to 15 words, the embedding vectors’ size to 768, and the number of training epochs to 20, until no further improvement on the validation set could be observed; convergence assessments are provided in Figure D2 of the Supporting Information (available at https://doi.org/10.1162/nol_a_00125).

### GPT-2 Training

GPT-2 (Generative Pretrained Transformer 2) is a deep learning transformer-based language model. We trained the open-source implementation GPT2LMHeadModel, provided by HuggingFace (Wolf, 2020), on the three datasets (integral, semantic, and syntactic).

The GPT2LMHeadModel architecture is trained on a next-token prediction task using a CrossEntropyLoss and the Pytorch Python package (Paszke et al., 2019). The training procedure can easily be extended to any feature type by adapting both vocabulary size and tokenizer to each vocabulary. Indeed, the inputs given to GPT2LMHeadModel are ids encoding vocabulary items. All the analyses reported in this article were performed with 4-layer models having 768 units per layer and 12 attention heads. As shown in Pasquiou et al. (2022), these 4-layer models fit brain data nearly as well as the usual 12-layer models. We presented the models with input sequences of 512 tokens and let the training run for five epochs until no further improvement on the validation set could be observed; convergence assessments are provided in Figure D1 in the Supporting Information.

For the GTP-2 trained on the semantic features, small modifications had to be made to the model architecture in order to remove all residual syntax. By default, GPT-2 encodes the absolute positions of tokens in sentences. When training GPT-2 on the semantic features, as word ordering might contain syntactic information, we had to make sure that position information could not be leveraged by means of its positional embeddings, yet keeping information about word proximity as it influences semantics. We modified the implementation so that the GPT-2 trained on semantic features follows these specifications (see Appendix C in the Supporting Information).

### Stimulus: *The Little Prince* Story

The stimulus used to obtain activations from humans and from NLP models was *The Little Prince* (de Saint-Exupéry, 1943) novella. Humans listened to an audiobook version, spliced into nine tracks that lasted approximately 11 min each (see Li et al., 2022). In parallel, NLP models were provided with an exact transcription of this audio-book, enriched with punctuation signs from the written version of *The Little Prince*. The text comprised 15,426 words and 4,482 punctuation signs. The acoustic onsets and offsets of the spoken words were marked to align the audio recording with the *The Little Prince* text.

### Computing Embeddings From *The Little Prince* Text

The tokenized versions of *The Little Prince* (one for each feature type) were run through GloVe and GPT-2 in order to generate embeddings that could be compared with fMRI data.

For GloVe, we simply retrieved the fixed embedding vector learned during training for each token.

For GPT-2, we retrieved the contextualized third layer hidden-state (aka embedding) vector for each token, so that the dimension is comparable to the dimension of GloVe’s embeddings (768 units). Layer 3 (out of 4) was selected because it has been demonstrated that late middle layers of recurrent language models are best able to predict brain activity (Jain & Huth, 2018; Toneva & Wehbe, 2019) (see Figure L2 in the Supporting Information).

The embedding built by GPT-2 for a given token rely on the past tokens (aka past context). The bigger the past context, the more reliable the token embedding will be. We designed the following procedure to ensure that the embedding of each token used similar past context size: the input sequence was limited to a maximum of 512 tokens. The text was scanned with a sliding window of size *N* = 512 tokens, and a step of 1 token. The embedding vector of the next to last token (in the sliding window) was then retrieved. For the context-constrained versions of GPT-2 (denoted GPT-2_Context−*k*_), the input text was formatted as the training data (see Figure 1C) in batches of input sequences of length (*k* + 5) tokens (see Appendix B in the Supporting Information for examples), and only the embedding vector of the current token was retrieved. Embedding matrices were built by concatenating words embeddings. More precisely, calling *d* the dimension of the embeddings retrieved from of a neural model, corresponding to the number of units in one layer in our case, and *w* the total number of tokens in the text, we obtained an embedding matrix **X** ∈ ℝ^*w*×*d*^ after the presentation of the entire text to the model.

### Decoding of Syntax and Semantics Categories From Embeddings

We designed two decoding tasks: a syntax decoding task in which we tried to predict the triplet (POS, Morph, NCN) of each word from its embedding vector (355 categories), and a semantic decoding task in which we tried to predict each *content word*’s semantic category (from Wordnet, https://wordnet.princeton.edu/) from its embedding vector (837 categories).

We used logistic classifiers and the text of *The Little Prince*, which was split using a ninefold cross-validation on runs, training on eight runs and evaluating on the remaining one for each split. Dummy classifiers were fitted and used as estimations of chance-level for each task and model. It is crucial to acknowledge that the baseline performance level varies based on both the decoding task and the specific model employed. Specifically, the models trained on semantic features were exclusively trained on content words. Consequently, when assessing the syntactic decoding accuracy of these models, only content words were considered, resulting in an elevated baseline performance level. Conversely, for the models trained on syntactic/integral features, the syntactic decoding accuracy encompasses the evaluation of all tokens. All classifiers implementations were taken from Scikit-Learn (Pedregosa et al., 2011).

### MRI Data

We used the fMRI data of 51 English speaking participants who listened to an entire audiobook of *The Little Prince* during about one hour and a half. These data, available at https://openneuro.org/datasets/ds003643/versions/1.0.2, are described in detail by Li et al. (2022). In short, the acquisition used echo-planar imaging (repetition time = 2 s; resolution = 3.75 × 3.75 × 3.75 mm) with a multi-echo (3 echos) sequence to optimize signal-to-noise (Kundu et al., 2017). Preprocessing comprised multi-echo independent components analysis to denoise data for motion, physiology and scanner artifacts, correction for slice-timing differences, and nonlinear alignment to the Montreal Neurological Institute (MNI) template brain.

For each participant, there were nine runs of fMRI acquisition representing about 10 min of brain activations each. We re-sampled the preprocessed individual scans at 4 × 4 × 4 mm (to reduce computation load) and applied linear detrending and standardization (mean removal and scaling to unit variance) to each voxel’s time series.

Finally, we computed a global brain mask to keep only voxels containing useful signal (using the compute_epi_mask function in nilearn (Thual et al., 2023), we find the least dense point of the total image histogram) across all runs for at least 50% of all participants. This global mask contained 26,164 voxels at 4 × 4 × 4 mm resolution. All analyses reported in this article were performed within this global mask.

### Correlations Between Embeddings and Individual fMRI Data

The embeddings (**X**) derived from neural language models were mapped to each subject’s fMRI activations (**Y***_s_*, *s* = 1 .. *S*) following the pipeline outlined in Figure 1B.

The process, using the standard model-based encoding approach to modeling fMRI signals (Huth et al., 2016; Naselaris et al., 2011; Pasquiou et al., 2022), is detailed in Appendix F in the Supporting Information. In brief, each column of **X** was first aligned with the words’ offsets in the audio stream and convolved with the default *SPM* haemodynamic kernel (using Nilearn’s *compute_regressor* function from the *nilearn.glm.first_level* module). The resulting time course was sub-sampled to match the sampling frequency of the scans **Y***_s_* (giving **~X**). Next, in each individual voxel, the time course of brain activation was regressed on **~X** using Ridge regression. The Ridge regression regularization was estimated using a nested-cross validation scheme (see Appendix F in the Supporting Information for more details). Finally, the cross-validated Pearson correlation *R* between the encoding model’s prediction and the fMRI signal for subject *s* in voxel *v* was computed. The output of this process is a map of correlations between the encoding models’ predictions and the observed time series, for a given participant.

### Baseline fMRI Model

To obtain a more accurate evaluation of the specific impact of the embeddings on brain scores, we removed the contribution of three confounding variables from all maps presented in this paper. The three confounding variables were (1) *the acoustic energy* (root mean squared of the audio signal sampled every 10 ms; (2) *the word-rate* (one event at each word offset; and (3) *the log of the unigram lexical frequency* of each word (modulator of the word events. An fMRI Ridge linear model that only included these three regressors was used to compute a map of cross-validated correlations for each participant.

In the rest of the paper, Δ*R* refers to the increase in *R* when adding a model to the baseline model versus the baseline model by itself.

Figure G1 in the Supporting Information displays the significant correlations in the group-level *R* maps associated with the baseline model, corrected for multiple comparison using a false discovery rate (FDR) correction (*p* < 0.005).

### Group-Level Maps

The brain maps presented in this article display group average increase in *R* scores obtained from individuals correlation maps (relative to the baseline model or to another model). Only voxels showing statistically significant increase in *R* score are shown.

Significance was assessed through one-sample *t* tests applied to the spatially smoothed correlation maps, with an isotropic Gaussian kernel with full width at half maximum (FWHM) of 6 mm. In each voxel, the test assessed whether the distribution of *R*_test_ values across participants was significantly larger than zero. To control for multiple comparisons, all maps were corrected using a FDR correction with *p* < 0.005 (Benjamini & Hochberg, 1995). On each corrected figure, the FDR threshold on the *z*-scores, named *z*_FDR_, is indicated at the bottom, that is, values reported on these maps (e.g., *R* scores) are shown only for voxels whose *z*-score survived this threshold (*z*_voxel_ > *z*_FDR_).

While all analyses were done on volume data, all brain maps were projected onto brain surface for visualization purposes, using ‘*fsaverage5*’ (from Nilearn’s *datasets.fetch_surf_fsaverage*) mesh and the ‘*vol_to_surf*’ function (from Nilearn’s *surface* module).

### Syntax and Semantics Peak Regions

We decided to also report brain maps’ *peak regions*, that is, the 10% of the voxels having the highest *R* score in a brain map. The motivation is that two different language processes might elicit lots of brain regions in common, while the regions that are better fitted by the representations derived from each process might differ. The peak regions of the neural correlates of semantic and syntactic representations are displayed on surface brain maps. The proportions of voxels belonging to each peak region as well as the Jaccard score between syntax and semantics are displayed for each model and hemisphere. Subject-level maps were added in the Supporting Information to complement our group-level analysis.

### Jaccard Index

The Jaccard index (computed using scikit-learn *jaccard_score* function from the *metrics* module) for two sets *X* and *Y* is defined in the following manner: *J*(*X*, *Y*) = ∣*X* ∩ *Y*∣ / ∣*X* ∪ *Y*∣. It behaves as a similarity coefficient: when the two sets completely overlap, J = 1; when their intersection is nil, J = 0.

### Specificity Index

To quantify how much each voxel *v* is influenced by semantic and syntactic embeddings, we defined a *specificity index* in the following manner:

$$x_{\text{specificity}}\left(v\right)=\log_{10}\left(\frac{r_{\text{Semantic}}\left(v\right)}{r_{\text{Syntax}}\left(v\right)}\right)$$

*r*_Syntax_ is the *R* score increase relative to the baseline model for the syntactic embeddings. *r*_Semantic_ is the *R* score increase relative to the baseline model for the semantic embeddings.

The higher *x* is, the more sensitive it is to semantic embeddings compared to syntactic embeddings. The lower *x* is, the more sensitive it is to syntactic embeddings compared to semantic embeddings. *x* close to 0 indicates an equal sensitivity to syntactic and semantic embeddings.

Group average specificity index maps were computed from each subject’s map and significance was assessed through one-sample *t* tests applied to the spatially smoothed specificity maps, with an isotropic Gaussian kernel with FWHM of 6 mm. A FDR correction (*p* < 0.005) was used to correct for multiple comparisons.

## RESULTS

### Dissociation of Syntactic and Semantic Information in Embeddings

We first assessed the amount of syntactic and semantic information contained in the embedding vectors derived from GloVe and GPT-2 trained on the different sets of features. In order to do so, we trained logistic classifiers to decode either the semantic category or the syntactic category from the embeddings generated from the text of *The Little Prince*.

**Figure 2.**
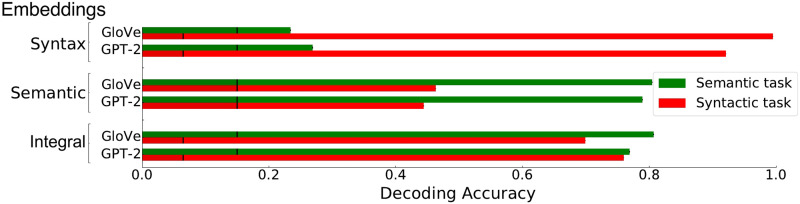
Decoding syntactic and semantic information from word embeddings. For each dataset and model type (GloVe and GPT-2), logistic classifiers were set up to decode either the syntactic or the semantic categories of the words from the text of *The Little Prince*. Chance level was assessed using dummy classifiers and is indicated by black vertical lines.

The decoding performances of the logistic classifiers are displayed in Figure 2. The models trained directly on the integral features, that is, the intact texts, have relatively high performance on the two tasks (75% in average for both GloVe and GPT-2). The models trained on the syntactic features performed well on the syntax decoding task (decoding accuracy >95%), but are near chance-level on the semantic decoding task (decoding accuracy around 25% with a chance-level at 16%). Similarly, the models trained on the semantic features display good performance on the semantic decoding task (decoding accuracy greater than 80%) but a relatively poorer decoding accuracy on the syntax decoding task (45%, chance level: 16%). These results validate the experimental manipulation by showing that syntactic embeddings essentially encode syntactic information and semantic embeddings essentially encode semantic information. The high decoding accuracy of GloVe models is to be expected as we are decoding fixed categories associated with each word. Most of the information contained in the syntactic label (POS + Morph) and the semantic label is independent of the context, thus, GloVe performs well because it ignores contextual information. On the other hand, GPT-2 may be slightly affected by contextual cues. Despite this, the decoding task remains useful in demonstrating the presence of specific information within a model’s embeddings.

In the Supporting Information, we present the decoding accuracy of the models when independently decoding the POS, Morph, and NCN. These findings reveal that models trained on semantic features perform at chance-level when predicting the NCN, while surpassing chance-level accuracy when predicting the Morph and POS. This improved performance in Morph prediction can be attributed to the retention of certain features such as gender, plural, or tense, which were preserved to maintain semantic integrity. POS is well decoded because of the small number of POS labels compared to the vocabulary size (number of content words).

### Correlations of fMRI Data With Syntactic and Semantic Embeddings

**Figure 3.**
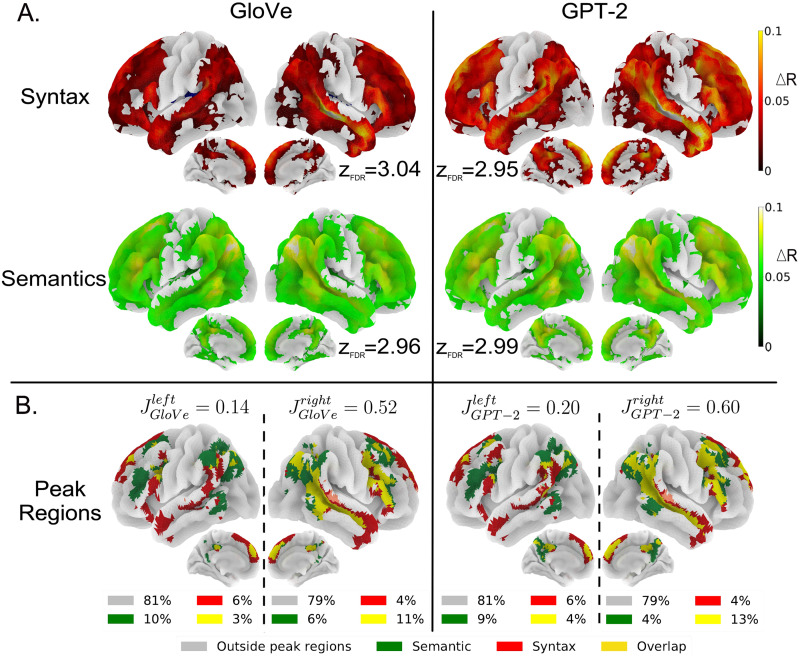
Comparison of the ability of GloVe and GPT-2 to fit brain data when trained on either the semantic or the syntactic features. (A) Significant increase in *R* scores relative to the baseline model for GloVe (a noncontextual model) and GPT-2 (a contextual model), trained either on the syntactic features or on the semantic features (voxel-wise thresholded group analyses; *N* = 51 subjects; corrected for multiple comparisons with a FDR approach *p* < 0.005; for each figure *z*_FDR_ indicates the significance threshold on the *Z* scores). (B) Bilateral spatial organization of syntax and semantics highest *R* scores. Voxels whose *R* scores belong in the 10% highest *R* scores (in green for models trained on the semantic features, and in red for models trained on the syntactic features) are projected onto brain surface maps for GloVe and GPT-2 (overlap in yellow and other voxels in gray). Jaccard score for each hemisphere are computed, i.e., the ratio between the size of the intersection and the size of the union of semantics and syntax peak regions; the proportion of voxels of each category are displayed for each hemisphere and model.

Our objective was to evaluate how well the embeddings computed from GloVe and GPT-2 on the syntactic and semantic features fit the fMRI signal in various parts of the brain. For each model/features combination, we computed the increase in *R* score when the resulting embeddings were appended to a baseline model that comprised low-level variables (acoustic energy, word onsets, and lexical frequency). This was done separately for each voxel. The resulting maps are displayed in Figure 3A.

The maps reveal that semantic and syntactic feature-derived embeddings from GloVe or GPT-2 significantly explain the signal in a set of bilateral brain regions including frontal and temporal regions, as well as the temporo-parietal junction (TPJ), the precuneus, and dorso-medial prefrontal cortex (dMPC). The classical left-lateralized language network, which includes the inferior frontal gyrus (IFG), and the superior temporal sulcus (STS), is entirely covered. Overall, a vast network of regions is modulated by both semantic and syntactic information.

Nevertheless, detailed inspection of the maps shows different *R* score distribution profiles (see Appendix I in the Supporting Information). For example, syntactic embeddings yield the highest fits in the superior temporal lobe, extending from the temporal pole (TP) to the temporo-parietal junction (TPJ), as well as the IFG (BA-44 and 47), the superior frontal gyrus (SFG), the dorso-medial prefrontal cortex (dMPC), and the posterior cingulate cortext (pCC). Semantic embeddings, on the other hand, show peaks in the posterior middle temporal gyrus (pMTG), the angular gyrus (AG), the inferior frontal sulcus (IFS), the dMPC, and the precuneus/pCC.

### Regions Best Fitted by Semantic or Syntactic Embeddings

As noticed above, despite the fact that the regions fitted by semantic and syntactic embeddings essentially overlap (Figure 3A), the areas where each model has the highest *R* scores differ. To better visualize the maxima from these maps, we selected, for each of them, the 10% of voxels having the highest *R* scores. Thresholding at the 90th percentile of the distributions (threshold values displayed in Figure I1 in the Supporting Information) produces the maps presented in Figure 3B.

A first observation is that the number of supra-threshold voxels is quite similar in the left (19%) and right (21%) hemispheres, whether GPT-2 or GloVe is considered, showing that during the processing of natural speech, both syntactic and semantic features modulate activations in both hemispheres to a similar extent. The regions involved include, bilaterally, the TP, the STS, the IFG and IFS, the dMPC, the pMTG, the TPJ, the precuneus, and pCC.

One noticeable difference between the two hemispheres, apparent in Figure 3B, concerns the overlap between the semantic and syntactic peak regions: It is stronger in the right than in the left hemisphere. To assess this overlap, we computed the Jaccard indexes (see Jaccard Index) between voxels modulated by syntax and voxels modulated by semantics. The Jaccard indexes were much larger in the right hemisphere ($J_{\text{GloVe}}^{\text{right}}$ = 0.52 and $J_{\text{GPT}-2}^{\text{right}}$ = 0.60) than in the left ($J_{\text{GloVe}}^{\text{left}}$ = 0.14 and $J_{\text{GPT}-2}^{\text{left}}$ = 0.20).

The left hemisphere displayed distinct peak regions for semantics and syntax; syntax involving the STS, the posterior superior temporal gyrus (STG), the anterior TP, the IFG (BA-44/45/47), and the middle front gyrus (MFG), whereas semantics involves the pMTG, AG, TPJ, and IFS. We only observe overlap in the upper IFG (BA-44), AG, and posterior STS. On medial faces, semantics and syntax share peak regions in the precuneus, the pCC, and the dMPC. In the right hemisphere, syntax and semantics share the STS, pMTG, and most frontal regions, with only syntax-specific peak regions in the TP and SFG and semantics-specific peak regions in the TPJ.

In addition to the group-level analysis, we conducted subject-level analyses that yielded consistent findings (see Figures K1–K3 in the Supporting Information). Our results demonstrate the following patterns:

- We observed higher Jaccard scores in the right hemisphere compared to the left.
- Syntactic peak regions were identified in the temporal regions, the IFG, and dMPFC.
- Semantic peak regions were found near the IFS, pMTG, and TPJ.

These subject-level analyses further support and reinforce the patterns observed at the group level.

Overall, this shows that the neural correlates of syntactic and semantic features appear more separable in the left than in the right hemisphere.

### Gradient of Sensitivity to Syntax or Semantics

The analyses presented above revealed a large distributed network of brain regions sensitive to both syntax and semantics but with varying local sensitivity to both conditions.

**Figure 4.**
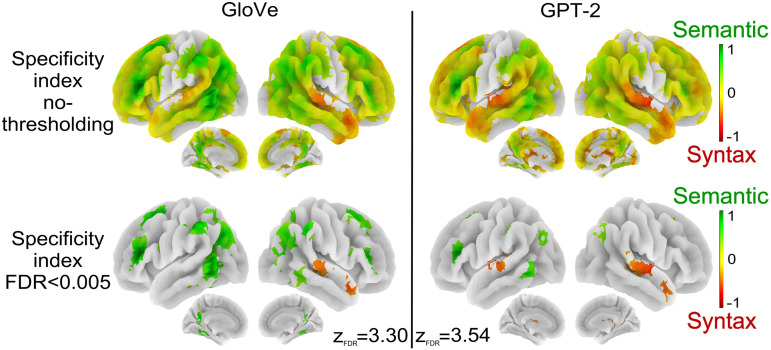
Voxels’ sensitivity to syntactic and semantic embeddings. Voxels’ specificity indexes are projected onto brain surface maps reflecting how much semantic information helps to better fit the time courses of a voxel compared to syntactic information; the greener the more the voxel is categorized as a semantic voxel, the redder the more the voxel is categorized as a syntactic voxel. Yellow regions are brain areas where semantic and syntactic information lead to similar *R* score increases. The top row displays specificity indexes in voxels where there was a significant effect for semantic or syntactic embeddings in Figure 3A. The bottom row is the voxel-wise thresholded group analyses; *N* = 51 subjects; corrected for multiple comparisons with FDR < 0.005 (for each figure *z*_FDR_ indicates the significance threshold on the *Z* scores).

We further investigated these differences by defining a specificity index that reflects, for each voxel, the logarithm of the ratio between the *R* scores derived from the semantic and the syntactic embeddings (see Specificity Index). A score of *x* indicates that the voxel is 10^*x*^ times more sensitive to semantics compared to syntax if *x* > 0 (green), and conversely, the voxel is 10^*x*^ times more sensitive to syntax compared to semantics if *x* < 0 (red). Voxels with specificity indexes close to 0 are colored in yellow and show equal sensitivity to both conditions. Specificity indexes are plotted on surface maps in Figure 4. The top row shows the specificity index of voxels where there was a significant effect for syntactic or for semantic embeddings in Figure 3A, while the bottom row shows group specificity indexes corrected for multiple comparison using an FDR-correction of 0.005 (*N* = 51).

The top row of Figure 4 shows that voxels that are more sensitive to syntax include, bilaterally, the anterior temporal lobes (aTL), the STG, the supplementary motor area (SMA), the MFG and sub-parts of the IFG. Voxels more sensitive to semantics are located in the pMTG, the TPJ/AG, the IFS, the SFS, and the precuneus. Voxels sensitive to both types of features are located in the posterior STG, the STS, the dMPC, the CC, the MFG, and the IFG.

More specifically, in Figure 4 bottom, one can observe significantly low ratios (in favor of the syntactic embeddings) in the STG, aTL, and pre-SMA, and significantly large ratios (in favor of the semantic embeddings) in the pMTG, the AG, and the IFS. Specificity index maps are consistent with the maps of *R* score differences between semantic and syntactic embeddings for GloVe and GPT-2 (see Figure J1 in the Supporting Information) but provide more insights into the relative sensitivity to syntax and semantics. These maps highlight that some brain regions show stronger responses to the semantic or to the syntactic condition even when they show sensitivity to both.

### Unique Contributions of Syntax and Semantics

The previous analyses allowed us to quantify the amounts of brain signal explained by the information encoded in various embeddings. Yet, when two embeddings explain the same amount of signal, that is, have similar *R* score, it remains to be clarified whether they hinge on information represented redundantly in the embeddings or information specific to each embedding. To address this issue, we analyzed the additional information brought by each embedding on top of the other one. To this end, we evaluated correlations that are uniquely explained by the semantic embeddings compared to the syntactic embeddings, and conversely.

To quantify the unique contribution of each feature space to the prediction of the fMRI signal, we first estimated the Pearson correlation explained by the embeddings learned from the individual feature space—for example, using only syntactic embeddings or semantic embeddings. We then assessed the correlation explained by the concatenation of embeddings derived from different feature spaces—e.g., concatenating syntactic and semantic embedding vectors (de Heer et al., 2017).

Because it can identify single voxels whose responses can be partly explained by different feature spaces, this approach provides more information than simple subtractive analyses that estimate the *R* score difference per voxel (see Figure J1 in the Supporting Information).

**Figure 5.**
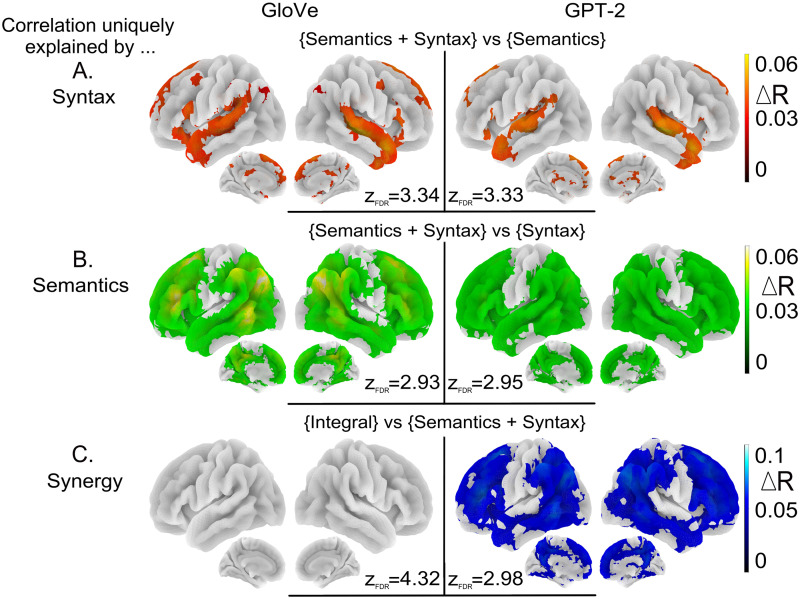
Correlation uniquely explained by each embedding. (A) Increase in *R* scores relative to the semantic embeddings when concatenating semantic and syntactic embeddings in the encoding model. (B) Increase in *R* scores relative to the syntactic embeddings when concatenating semantic and syntactic embeddings in the encoding model. (C) Increase in *R* scores relative to the concatenated semantic and syntactic embeddings for the integral embeddings. These maps are voxel-wise thresholded group analyses; *N* = 51 subjects; corrected for multiple comparisons with a FDR approach *p* < 0.005; for each figure *z*_FDR_ indicates the significance threshold on the *Z* scores.

Syntactic embeddings (Figure 5A) uniquely explained brain data in localized brain regions: the STG, the TP, pre-SMA, and IFG, with *R* score increases of about 5%.

Semantic embeddings (Figure 5B) uniquely explained signal bilaterally in the same wide network of brain regions as the one highlighted in Figure 3A, including frontal and temporo-parietal regions bilaterally as well as the precuneus and pCC medially, with similar *R* score increases around 5%.

This suggests that even if most of the brain is sensitive to both syntactic and semantic conditions, syntax is preferentially processed in more localized regions than semantics, which is widely distributed.

### Synergy Between Syntax and Semantics

To probe regions where the joint effect of syntax and semantics is greater than the sum of the contributions of these features, we compared the *R* scores of the embeddings derived from the integral features with the *R* scores of the encoding models concatenating the semantic and syntactic embeddings (see Figure 5C).

For the embeddings obtained with GloVe, this analysis did not reveal any significant effect. For the embeddings obtained with GPT-2, significant effects were observed in most of the brain, but with higher effects in the semantic peak regions (pMTG, TPJ, AG) and in frontal regions.

### Integration of Contextual Information

**Figure 6.**
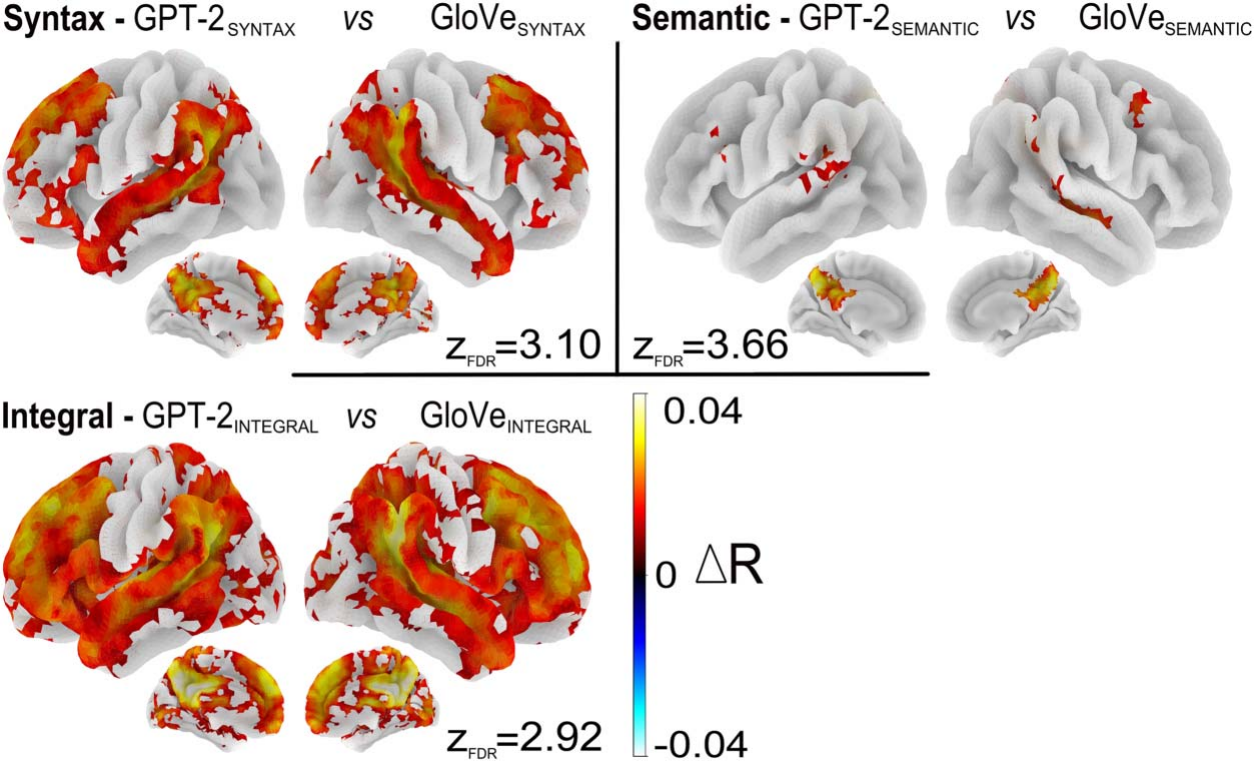
Comparison of lexical and supra-lexical processing levels. Brain regions that are significantly better predicted by GPT-2 (in red) compared to GloVe, when trained on syntactic features (top left), semantic features (top right), and integral features (bottom left). Maps are voxel-wise thresholded group analyses; *N* = 51 subjects; corrected for multiple comparisons with a FDR approach *p* < 0.005; for each figure *z*_FDR_ indicates the significance threshold on the *Z* scores.

To further examine the effect of context, we compared GPT-2, the supra-lexical model which takes context into account, to GloVe, a purely lexical model. The differences in *R* scores between the two models, trained on each of the three datasets are presented in Figure 6.

GPT-2 embeddings elicit stronger *R* scores than GloVe. The difference spreads over wider regions when the models are trained on syntax compared to semantics (see Figure 6 top left and right). The comparison for syntax led to significant differences bilaterally in the STS/STG, from the TP to the TPJ, in superior, middle and inferior frontal regions, and medially in the pCC and dMPC. For semantics, the comparison only led to significant differences in the Precuneus, the right STS, and posterior STG. Figure 6 (bottom left) shows the comparison between GPT-2 and GloVe when trained on the integral features. Given that both semantic and syntactic contextual information were available to GPT-2, these maps reflect the regions that benefit from context during story listening.

To show that context has an effect is one thing, but different brain regions are likely to have different integration window sizes. To address this question, we developed a fixed-context window training protocol to control for the amount of contextual information used by GPT-2 (Figure 1C). We trained models with short (5 tokens), medium (15 tokens), and long (45 tokens) range window sizes. This ensures that GPT-2 was not sampling out of the learned distribution at inference, and not using more context than what was available in the context window.

**Figure 7.**
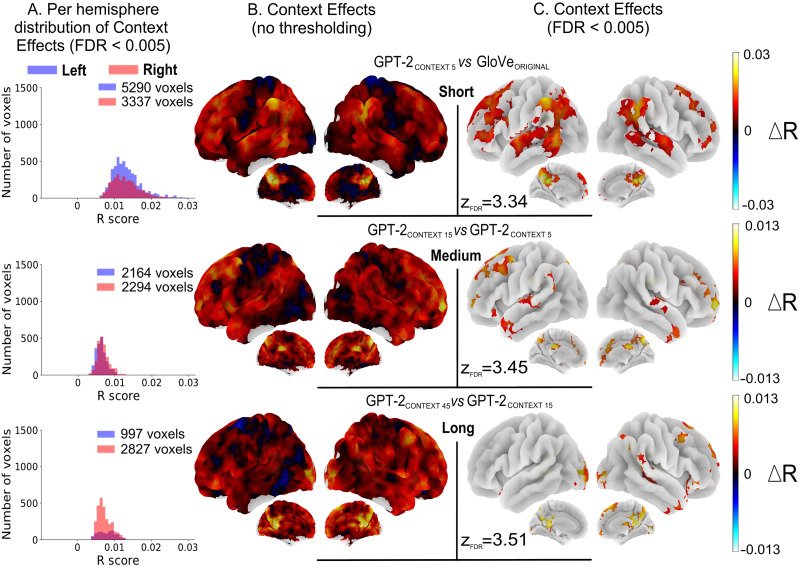
Integration of context at different levels of language processing. (A) Per hemisphere histograms of significant context effects after group analyses (*N* = 51 subjects); thresholded at *p* < 0.005 voxel-wise, corrected for multiple comparisons with the FDR approach. (B) Uncorrected group averaged surface brain maps representing *R* score increases when fitting brain data with models leveraging increasing sizes of contextual information. (C) Corrected group averaged surface brain maps representing *R* scores increases when fitting brain data with models leveraging increasing sizes of contextual information; thresholded at *p* < 0.005 voxel-wise, corrected for multiple comparisons with the FDR approach (for each figure *z*_FDR_ indicates the significance threshold on the *Z* scores). (Top row) Comparison of the model trained with 5 tokens of context (GPT-2_Context−5_) with the noncontextualized GloVe. (Middle row) Comparison of the models, respectively, trained with 15 (GPT-2_Context−15_) and 5 (GPT-2_Context−5_) tokens of context. (Bottom row) Comparison of the models, respectively, trained with 45 (GPT-2_Context−45_) and 15 (GPT-2_Context−15_) tokens of context.

Comparing GPT-2 with 5 tokens to GloVe (0-size context) highlighted a large network of frontal and temporo-parietal regions. Medially, it included the precuneus, the pCC, and the dMPC (Figure 7, Short). Short context-sensitivity showed peak effects in the supramarginal gyri, the pMTG, and medially in the precuneus and pCC. Counting the number of voxels showing significant short-context effects highlighted an asymmetry between the left and right hemisphere with 1.6 times more significant voxels in the left hemisphere compared to the right. Contrasting a GPT-2 model using 15 tokens of context (the average size of a sentence in *The Little Prince*) versus a GPT-2 model using only 5 tokens, yielded localized significant differences in the SFG/SFS, the TP, MFG, and STG near Heschl’s gyri, and medially in the precuneus and pCC (Figure 7, Medium). The biggest medium context effects included the left MFG, the right SFG and dMPC, and bilaterally the precuneus and pCC. Finally, contrasting models using, respectively, 45 and 15 tokens of context revealed 2.8 times as many significant differences in the right hemisphere as in the left. Significant effects were the highest bilaterally and medially in the pCC, followed, in the right hemisphere, by the precuneus, dMPC, MFG, SFG, STS, and TP (see Figure 7, bottom).

Taken together, our results show (1) that syntax dominantly determines the integration of contextual information, (2) that a bilateral network of frontal and temporo-parietal regions is modulated by short context, (3) that short-range context integration is preferentially located in the left hemisphere, (4) that the right hemisphere is involved in the processing of longer context sizes, and finally (5) that medial regions (precuneus and pCC) are core regions of context integration, showing context effects at all scales.

## DISCUSSION

Language comprehension in humans is a complex process, which involves several interacting subcomponents (e.g., word recognition, processing of syntactic and semantic information to construct sentence meaning, pragmatic and discourse inference; Jackendoff, 2002). Discovering how the brain implements these processes is one of the major goals of neurolinguistics. A lot of attention has been devoted, in particular, to the syntactic and semantic components (Binder & Desai, 2011; Friederici, 2017, for reviews), and the extent to which they are implemented in distinct or identical regions is still debated (e.g., Fedorenko et al., 2020).

It must be noted that a fair proportion of these studies relied on controlled experimental paradigms with single words or sentences, based on the manipulation of complexity or violations of expectations. To study language processing in a more natural way, several recent studies have presented naturalistic texts to participants, and have analyzed their brain activations using artificial neural language models (e.g., Huth et al., 2016; Pasquiou et al., 2022; Pereira et al., 2018; Schrimpf et al., 2021). These models are known to encode some aspects of semantics and syntax (e.g., Hewitt & Manning, 2019; Lakretz et al., 2019; Pennington et al., 2014). In the current work, to further dissect brain activations into separate linguistic processes, we trained NLP models on a corpus from which we selectively removed syntactic, semantic, or contextual information and examined how well these information-restricted models could explain fMRI signal recorded from participants who had listened to an audiobook. The rationale was to highlight brain regions representing syntactic and semantic information, at the lexical and supralexical levels (comparing a lexical model, GloVe, and a contextual one, GPT-2). Additionally, by varying the amount of context provided to the supralexical model, we sought to identify the brain regions sensitive to different context sizes (see Jain & Huth, 2018, for a similar approach).

Whether models were trained on syntactic features or on semantic features, they fit fMRI activations in a wide bilateral network that goes beyond the classic language network comprising the IFG and temporal regions: It also includes most of the dorso lateral and medial prefrontal cortex, the inferior parietal cortex, and on the internal face, the precuneus and pCC (see Figure 3). Nevertheless, the regions best predicted by syntactic features on the one hand, and semantic features on the other hand, are not exactly the same. While they overlap quite a lot in the right hemisphere, they are more dissociated in the left hemisphere. See Figure 3B, above, and Figure K1 in the Supporting Information. In addition, the relative sensitivity to syntax and semantics varies from region to region, with syntax predominating in the temporal lobe (see Figure 4). Elimination of shared variance between syntactic and semantic features confirmed that pure syntactic effects are restricted to STG/STS, bilaterally, IFG, and pre-SMA, while pure semantic effects occur throughout the network (Figure 5A–B).

The comparison between the supralexical model (GPT-2) and the lexical one (GloVe), revealed brain regions involved in compositionality (Figure 6) and a synergy between syntax and semantics that arises only at the supralexical level (Figure 5C). Finally, analyses of the influence of the size of context provided to GPT-2 when computing word embeddings show that (1) a bilateral network of fronto-temporo-parietal regions is sensitive to short context; (2) there is a dissociation between the left and right hemispheres, respectively, associated with short-range and long-range context integration; and (3) the medial precuneus and posterior cingulate gyri show the highest effects at every scale, hinting at an important role in large context integration (Figure 7).

In summary, this study shows that

- there is a difference between the right and left hemispheres with respect to the separation of syntactic and semantic processing. We found more segregation in the left compared to the right hemisphere. This provides support to classic theories on the functional difference between the left and right (Beeman & Chiarello, 2013).
- the right hemisphere is sensitive to longer contexts than the left one (Beeman & Chiarello, 2013).
- neural language models are a beneficial tool in the study of brain function. Manipulating the training corpus or the size of the context window, possible only in simulations, was shown to lead to new findings about language processing in the human brain.

### Models Trained on Semantic and Syntactic Features Fit Brain Activity in a Widely Distributed Network, but With Varying Relative Degrees

When trained on the integral corpus, that is, on the integral features, both the lexical (GloVe) and contextual (GPT-2) models captured brain activity in a large *extended language network* (see Appendix H in the Supporting Information). This large extended language network goes beyond the core language network, that is, the left IFG and temporal regions, encompassing homologous areas in the right hemisphere, the dorsal prefrontal regions, both on the lateral and medial surfaces, as well as in the inferior parietal, precuneus and posterior cingulate. The result is consistent with the ones from previous studies that have looked at brain responses to naturalistic text, whether analysed with NLP models (e.g., Caucheteux et al., 2021; Huth et al., 2016; Jain & Huth, 2018; Pereira et al., 2018) or not (Chang et al., 2022; Lerner et al., 2011).

The precuneus/pCC, dMPC, and inferior parietal cortex are part of the default mode network (DMN; Raichle, 2015). The same areas are actually also relevant in language and high-level cognition. For example, early studies examining the role of coherence during text comprehension had pointed out the same regions (Ferstl & von Cramon, 2001; Xu et al., 2005): coherent discourses elicit stronger activations than incoherent ones. Recent work by Chang et al. (2022) has revealed that the DMN is the last stage in a temporal hierarchy of processing naturalistic text, integrating information on the scale of paragraphs and narrative events (see also Baldassano et al., 2017; Simony et al., 2016). These regions are not language-specific though, as they have been shown to be activated during various theory of mind tasks, relying on language or not, and have thus also been dubbed the mentalizing network (Baetens et al., 2014; Mar, 2011).

Models trained on the information-restricted semantic and syntactic features fit signal in this widely distributed network (Figure 3A). This is in agreement with Caucheteux et al. (2021) and Fedorenko et al. (2020), who, using very different approaches, found that syntactic predictors modulated activity throughout the language network. Caucheteux et al. (2021) first constructed new texts that matched, as well as possible, the text presented to participants in terms of their syntactic properties. The lexical items being different, the semantics of the new texts bear little relation with the original text. Then, using a pre-trained version of GPT-2, the authors obtained embeddings from these new texts and averaged them to create syntactic predictors. They found that these syntactic embeddings fitted a network of regions (Caucheteux et al., 2021, Figure 5D) similar to the one we observed (Figure 3A). Further, defining the effect of semantics as the difference between the scores obtained from the embeddings from the original text and the scores from the syntactic embeddings, Caucheteux et al. (2021) observed that semantics had a significant effect throughout the same network (Caucheteux et al., 2021, Figure 5D).

Should one conclude that syntax and semantics equally modulate the entire language network? Our results reveal a more complex picture. Figure 4 presents a semantics versus syntax specificity index map, showing higher sensitivity to syntax in the STG and aTL, whereas the parietal regions are more sensitive to semantics, consistent with Binder et al. (2009).

Our study helps to reconcile two apparently contradicting results in the literature. On the one hand, classic results on syntactic processing found a localized set of brain regions involved in syntactic processing (Friederici, 2016; Matchin et al., 2017; Pallier et al., 2011), whereas recent studies, using naturalistic (ecological) paradigms, found a more widely spread, distributed, network of brain regions involved in syntactic processing (Caucheteux et al., 2021; Fedorenko et al., 2020). Our study reconciles these two apparently contradicting results by providing a more graded view of syntactic processing in the brain, showing that sensitivity to syntactic processing peaks at around the same set of localized brain regions identified in classic studies.

Another point to take into consideration is that syntactic and semantic features are not perfectly orthogonal. Indeed, the logistic decoder trained on the embeddings from the semantic dataset was better than chance at recovering both syntactic Morphs and the POS (Figure 2, above, and Figure E1 in the Supporting Information). This might be due, for example, to the fact that some features like gender or number are present in both datasets, explicitly in the syntactic dataset and implicitly in the semantic dataset. POS can be easily decoded from semantic features because the number of POS labels is much smaller than the vocabulary size of the semantic features. To focus on the unique contributions of syntax and semantics, we remove the shared variance from the syntactic and semantic models using model comparisons (Figure 5).

### “Pure” Semantic but Not “Pure” Syntactic Features Modulate Activity in a Wide Set of Brain Regions

The unique effect of semantics, when its shared component with syntax was removed, remains widespread (Figure 5B). This is consistent with the notion that semantic information is widely distributed over the cortex, an idea popularized by embodiment theories (Hauk et al., 2004; Pulvermüller, 2013) but which was already supported by the neuropsychological observations revealing domain-specific semantic deficits in patients (Damasio et al., 2004).

On the other hand the “pure” effect of syntax “shrunk” to the STG and aTL (bilaterally), the IFG (on the left), and the pre-SMA (Figure 5A). The left IFG and STG/STS have previously been implicated in syntactic processing (e.g., Friederici, 2011, 2017), and this is confirmed by the new approach employed here. Note that we are not claiming that these regions are specialized for syntactic processing only. Indeed they also appear to be sensitive to the pure semantic component (Figure 5B).

### The Contributions of the Right Hemisphere

A striking feature of our results is the strong involvement of the right hemisphere. The notion that the right hemisphere has some linguistic abilities is supported by the studies on split-brains (Sperry, 1961) and by the patterns of recovery of aphasic patients after lesions in the left hemisphere (Dronkers et al., 2017). Moreover, a number of brain imaging studies have confirmed the right hemisphere involvement in higher-level language tasks, such as comprehending metaphors or jokes, generating the best endings to sentences, mentally repairing grammatical errors, detecting story inconsistencies (see Beeman & Chiarello, 2013; Jung-Beeman, 2005). All in all, this suggests that the right hemisphere is apt at recognizing distant relations between words. This conclusion is further reinforced by our observation of long-range (paragraph-level) context effects in the right hemisphere (Figure 7, long).

The effects we observed in the right hemisphere are not simply the mirror image of the left hemisphere. Spatially, syntax and semantics dissociate more in the left than the right. An observation that is consistent both at subject-level (see Figure K1 in the Supporting Information) and group-level (see Figure 3B). Moreover, the regions of overlap correspond to the regions integrating long context (Figure 7C, bottom row), suggesting that the left hemisphere is relatively more involved in the processing of local semantic or syntactic information, whereas the right hemisphere integrates both information at a larger time scale (supra-sentential).

### Syntax Drives the Integration of Contextual Information

The comparison between the predictions of the integral model trained on the intact texts and the predictions of the combined syntactic and semantic embeddings from the information-restricted models (Figure 5C), highlights a striking contrast between GloVe and GPT2. While the former, a purely lexical model, does not benefit from being trained on the integral text, GPT-2 shows clear synergetic effects of syntactic and semantic information. GPT-2’s embeddings fit brain activation better when syntactic and semantic information can contribute together. The fact that the regions that benefit most from this synergetic effect are high-level integrative regions, at the end of the temporal processing hierarchy described by Chang et al. (2022), suggests that the availability of syntactic information drives the semantic interpretation at the sentence level.

These regions are quite similar to the semantic peak regions highlighted in Figure 3B, and overlap with the regions showing context effects (Figure 7). This replicates, and extends, the results from Jain and Huth (2018), who, varying the amount of context fed to LSTM models, from 0 to 19 words, found shorter context effects in temporal regions (Jain & Huth, 2018, Figure 4).

It is crucial to clarify that the influence of syntactic information on semantic interpretation at the sentence level does not imply that syntactic information drives the alignment performance between artificial and biological neural networks. By examining the sensitivity index maps and comparing models trained on semantic features with those trained on syntactic features (see Figure J1 in the Supporting Information), it becomes apparent that models trained on semantic features account for a larger proportion of variance in most areas of the brain. The finding that semantic information accounts for a greater proportion of variance than syntactic information aligns with previous studies in the literature (Kauf et al., 2023; Mollica et al., 2020; Sinha et al., 2021).

### Limitations of Our Study

Two limitations of our study must be acknowledged.

The dissociation between syntax and semantics is not perfect. The way we created the semantic dataset by removing function words clearly impacts supra-lexical semantics. For example, removing instances of “and” and “or” prevents the NLP model from distinguishing between the meaning of “A or B” and “A and B.” In other words, the logical form of sentences can be perturbed. The decline in compositional semantics becomes apparent when examining the layer-wise encoding performance of the semantic model (see Figure L1 in the Supporting Information). In contrast to the models trained on integral or syntactic features, which exhibit optimal encoding performance in the later layers, the semantic model demonstrates a decrease in performance. This observation indicates that the model struggles to effectively utilize the structural information necessary for composing the meanings of more extensive linguistic structures.

This may partly explain the synergetic effect of syntax and semantics described above. Removing pronouns is also problematic as this removed the arguments of some verbs. Ideally, one would like to find transformations of the sentences that keep the semantic information associated to the function words, like conjunctions or pronouns, but it is not clear how to do that.

A second limitation concerns potential confounding effects of prosody. One cannot exclude that the embeddings of the models captured some prosodic variables correlated with syntax (Bennett & Elfner, 2019). For example, certain categories of words (e.g., determiners or pronouns) are shorter and less accented than others. Also, although the models are purely trained on written text, they acquire the capacity to predict the end of sentences, which are more likely to be followed by pauses in the acoustic signal. We included acoustic energy and the words’ offsets in the baseline models to try and diminish the impact of such factors, but such controls cannot be perfect. One way to address this issue would be to have participants read the text, presented at a fixed presentation rate. This would effectively remove all low-level effects of prosody.

## CONCLUSION

State-of-the-art NLP models, like transformers, trained with large enough corpora, can generate essentially flawless grammatical text, showing that they can acquire most of the grammar of the language. Using them to fit brain data has become a common endeavour, even if their architecture rules them out of plausible models of the brain. Yet, despite their low biological plausibility, their ability to build rich distributed representations can be exploited to study language processing in the brain. In this article, we have demonstrated that restricting information provided to the model during training can be used to show which brain areas encode this information. Information-restricted models are powerful and flexible tools to probe the brain as they can be used to investigate whatever representational space chosen, such as semantics, syntax, or context. Moreover, once they are trained, these models can be used directly on any dataset in order to generate information-restricted features for model-brain alignment. This approach is highly beneficial, both in term of richness of the features and scalability, compared to classical approaches that use manually crafted features or focus on specific contrasts. In future experiments, more fine-grained control of both the information given to the models as well as model’s representations will permit more precise characterization of the role of the various regions involved in language comprehension.

## ACKNOWLEDGMENTS

The authors acknowledge the use of the computing resources of the French Alternative Energies and Atomic Energy Commission (CEA) NeuroSpin research center.

## FUNDING INFORMATION

Bertrand Thirion, National Science Foundation (https://dx.doi.org/10.13039/100000001), Award ID: 1607441. Bertrand Thirion, Agence Nationale de la Recherche (https://dx.doi.org/10.13039/501100001665), Award ID: ANR-14-CERA-0001. Bertrand Thirion, HORIZON EUROPE Framework Programme (https://dx.doi.org/10.13039/100018693), Award ID: 945539 (Human Brain Project SGA3). Bertrand Thirion, KARAIB AI chair, Award ID: ANR-20-CHIA-0025-01.

## AUTHOR CONTRIBUTIONS

**Alexandre Pasquiou**: Conceptualization: Lead; Data curation: Lead; Formal analysis: Lead; Investigation: Lead; Methodology: Lead; Software: Lead; Validation: Lead; Visualization: Lead; Writing – original draft: Lead; Writing – review & editing: Lead. **Yair Lakretz**: Validation: Supporting; Writing – original draft: Supporting; Writing – review & editing: Equal. **Bertrand Thirion**: Funding acquisition: Lead; Project administration: Equal; Supervision: Equal; Validation: Supporting; Writing – original draft: Supporting; Writing – review & editing: Supporting. **Christophe Pallier**: Conceptualization: Equal; Data curation: Equal; Funding acquisition: Equal; Project administration: Lead; Supervision: Lead; Validation: Supporting; Writing – original draft: Equal; Writing – review & editing: Equal.

## DATA AVAILABILITY STATEMENT

The Integral Dataset (train, test, and dev) is available at: https://osf.io/jzcvu/. The semantic and syntactic datasets can be derived from the Integral Dataset using the scripts provided in https://github.com/AlexandrePsq/Information-Restricted-NLMs.

All analyses, as well as model training, features extraction and the fitting of encoding models were performed using Python 3.7.6 and can be replicated using the code provided in the same Github repository (https://github.com/AlexandrePsq/Information-Restricted-NLMs). The required packages are listed there. A non-exhaustive list includes numpy (Harris et al., 2020), scipy (Virtanen et al., 2020), scikit-learn (Pedregosa et al., 2011), matplotlib (Hunter, 2007), pandas (McKinney, 2010), and nilearn (https://nilearn.github.io/stable/index.html).

The fMRI dataset is publicly available at https://openneuro.org/datasets/ds003643/versions/1.0.2, and all details regarding the dataset are described in detail by Li et al. (2022).

## TECHNICAL TERMS

Semantics: The meaning of words, phrases, and sentences.
Language model: A model that assigns probabilities to sequences of words.
Syntax: The rules for structuring phrases and sentences in a language.
Contextual information: Knowledge gained from the previous words/tokens.
Features: Attributes/properties encoded by vectors.
Encoding model: A model that fits a set of features onto brain activity.
Supra-lexical: Above the individual word level, dealing with phrases/sentences.
Network of brain regions: Interconnected brain areas that work together.
Neurolinguistics: The study of how language is represented and processed in the brain.
