# Supplementary material for: Information-Restricted Neural Language Models Reveal Different Brain Regions’ Sensitivity to Semantics, Syntax, and Context
Source: Neurobiol Lang (Camb). 2023 Dec 14;4(4):611–36. doi: 10.1162/nol_a_00125 (PMC10745090; doi:10.1162/nol_a_00125)
Supplement: Supplementary file 1 [file nol-4-4-611-s001.pdf]

# Appendix A

## Models training

We trained GloVe and GPT-2 on syntactic or semantic features by adapting both vocabulary size and the associated tokenizer. Table A1 recapitulates information about the training of the models used. Table A2 provides examples of the features extracted from a short passage. After feature extraction, a vocabulary listing all possible feature instances is created for each feature type. A unique id is then associated to each element of the vocabulary. The tokenizer converts each feature to its unique id. Finally, the model is fed sequences of ids and learns to perform its task.

**Table A1**

*Models hyperparameters*

| **Models Models** | **Number of tokens** | **Number of unique words** | **Context window** | **Vector size** | **Number of epochs** | **Number of layers** |
|---|---|---|---|---|---|---|
| **GloVe Syntax** | 980 M | 1190 | 15 | 768 | 20 | NaN |
| **GPT-2 Syntax** | 980 M | 1190 | 512 | 768 | 5 | 4 |
| **GloVe Semantics** | 370 M | 91880 | 15 | 768 | 20 | NaN |
| **GPT-2 Semantics** | 370 M | 91880 | 512 | 768 | 5 | 4 |
| **GloVe Integral** | 980 M | 92945 | 15 | 768 | 20 | NaN |
| **GPT-2 Integral** | 980 M | 92945 | 512 | 768 | 5 | 4 |

**Table A2**

*Examples of input sequences given to the neural language models when trained on the different feature spaces.*

<table>
<tr><th></th><th></th><th colspan="7">Input sequence</th></tr>
<tr><td>Integral Features</td><td></td><td>The</td><td>sixth</td><td>planet</td><td>was</td><td>ten</td><td>times</td><td>larger</td></tr>
<tr><td rowspan="3">Syntactic Features</td><td>Part-of-Speech</td><td>DET</td><td>ADJ</td><td>NOUN</td><td>VERB</td><td>NOUN</td><td>NOUN</td><td>ADJ</td></tr>
<tr><td>Morphology</td><td>Definite=Def|PronType=Art</td><td>Degree =Pos</td><td>Number =Sing</td><td>Ind|Sing|Past| Person=3|Fin</td><td>Number =Card</td><td>Number =Plur</td><td>Degree =Cmp</td></tr>
<tr><td>Number of Closing Nodes</td><td>1</td><td>1</td><td>2</td><td>1</td><td>1</td><td>2</td><td>2</td></tr>
<tr><td>Semantic Features</td><td>Content words</td><td>–</td><td>sixth</td><td>planet</td><td>–</td><td>ten</td><td>times</td><td>larger</td></tr>
</table>

The Morphology field contains a list of morphological features, with vertical bar (|) as list separator and with underscore to represent the empty list. All features represent attribute-value pairs, with an equals sign (=) separating the attribute from the value. In addition, features are selected from the universal feature inventory (https://universaldependencies.org/u/feat/index.html) and are sorted alphabetically by attribute names. It is possible that a feature has two or more values for a given word: Case=Acc,Dat. In this case, the values are sorted alphabetically.

Note: for display purposes, the morphology attribute values were removed for 'was', it was originally equal to 'Mood=Ind|Number=Sing|Person=3|Tense=Past|VerbForm=Fin'.

## Appendix B

### Context-limited models

Using the same original collection of English novels from Project Gutenberg, we trained three GPT-2 models to probe context integration. More precisely, we restricted the preceding context (size $k = 5, 15$ or 45 tokens) given to the GPT-2 models during training on the "*Integral dataset*".

When training GPT-2 with a limited amount of contextual information, each input sequence contained $k + 5$ tokens: a special token at the beginning, $k$ context tokens, the current token for which we retrieve the activations in order to fit fMRI brain data, the token that is predicted by the current token and the 2 special tokens at the end (the last special end-of-sentence token is always preceded by a token encoding a blank space, we omitted it in the following table).

**Table B1**

*Examples of context-limited input sequences given to GPT-2 for the analyses on context-integration. Here the context size k is equal to 5.*

| Special token | Context (size = 5 tokens) | | | | | Current token | Predicted token | Special token |
|---|---|---|---|---|---|---|---|---|
| \|<endoftext>\| | Once | , | when | I | was | six | years | \|<endoftext>\| |
| \|<endoftext>\| | , | when | I | was | six | years | old | \|<endoftext>\| |
| \|<endoftext>\| | when | I | was | six | years | old | , | \|<endoftext>\| |
| \|<endoftext>\| | I | was | six | years | old | , | I | \|<endoftext>\| |

# Appendix C

## Removing absolute position information in GPT-2 trained on semantic features

For the GTP-2 model trained on the semantic features, small modifications had to be made to the model architecture in order to remove all residual syntax. By default, GPT-2 encodes the absolute positions of tokens in sentences. As word ordering might contain syntactic information, we had to make sure that it could not be leveraged by GPT-2 by means of its positional embeddings, yet keeping information about word proximity as it influences semantics. We achieved it by slightly modifying the architecture of GPT-2: we first removed the default positional embeddings, and added to the attention scores embeddings encoding relative positions between input tokens. Indeed, just removing positional embeddings would have led to a bag-of-words model. By adding these embeddings encoding relative position to the attention scores a token will weight the attention granted to another token depending on their distance. By doing so, information about absolute and relative positions is removed from tokens' embeddings as it is not directly added to the tokens' hidden states. The following explains how this operation was performed. Let $\mathbf{c}_W = (c_{w_1}, . . . , c_{w_m})$ be a sequence of $m$ tokenized content words. $\mathbf{c}_W$ is then fed to a $n_{layers}$ transformer with $n_{heads}$ of dimension $d_{heads}$ that first build an embedding representation $\mathbf{E}_i, i = 1..m$ (of size $d = d_{heads} * n_{heads}$) to which it appends (by default) a position embedding $\mathbf{p}_i, i = 1..m$ (of size $d$) for each token. To remove all syntactic content, the first step is to discard the previously mentioned positional embeddings $\mathbf{p}_i, i = 1..m$. However stopping here would only lead to a bag-of-word model where a given token might be influenced similarly by an adjacent token or one far away. As a consequence, we had to weight the attention score granted to a token depending on its relative distance.

The attention operation can be described as mapping a query (Q) and a set of key-value (K, V) pairs to an output, where the query, keys, values, and output are all vectors (generally packed into matrices). The output is computed as a weighted sum of the values, where the weight assigned to each value is computed by a compatibility function of

the query with the corresponding key. We thus modify the classical attention operation:

$$\text{Attention}(\mathbf{Q}, \mathbf{K}, \mathbf{V}) = \text{Softmax}((\mathbf{Q}\mathbf{K}^T)/\sqrt{d_k})\mathbf{V}$$

by adding the previously described relative positional embedding $\mathbf{W}$ in the attention mechanisms:

$$\text{Attention}(\mathbf{Q}, \mathbf{K}, \mathbf{V}) = \text{Softmax}((\mathbf{Q}\mathbf{K}^T + \mathbf{W})/\sqrt{d_k})\mathbf{V}$$

To build $\mathbf{W}$, we first defined the matrix $\mathbf{D} = (n - 1 + j - i)_{i,j=1..m} \in \mathbb{R}^{m \times m}$ (encoding the number of tokens separating two tokens in the input sequence shifted by $n - 1$) for each input sequence $\mathbf{c}_W$, where $n$ is the maximal input size. $\mathbf{D}$ is then embedded using a lookup table that stores an embedding of size $(d_{head})$ for each possible value of $\mathbf{D}$, giving $\mathbf{U}$ ($\in \mathbb{R}^{m \times m \times d_{head}}$).

Finally, the weights assigned to the value vectors are adjusted using the embedded relative distances between tokens $\mathbf{W}$ ($\in \mathbb{R}^{n_{heads} \times m \times m}$), defined as:

$$W_{i,j,k} = \sum_{d=1}^{d_{head}} K_{i,j,d} U_{j,k,d}$$

By doing so, we were able to weight words interactions depending on their relative distance in the input sequence, while removing all absolute positional information from tokens hidden-states.

## Appendix D

### Convergence of the language models during training

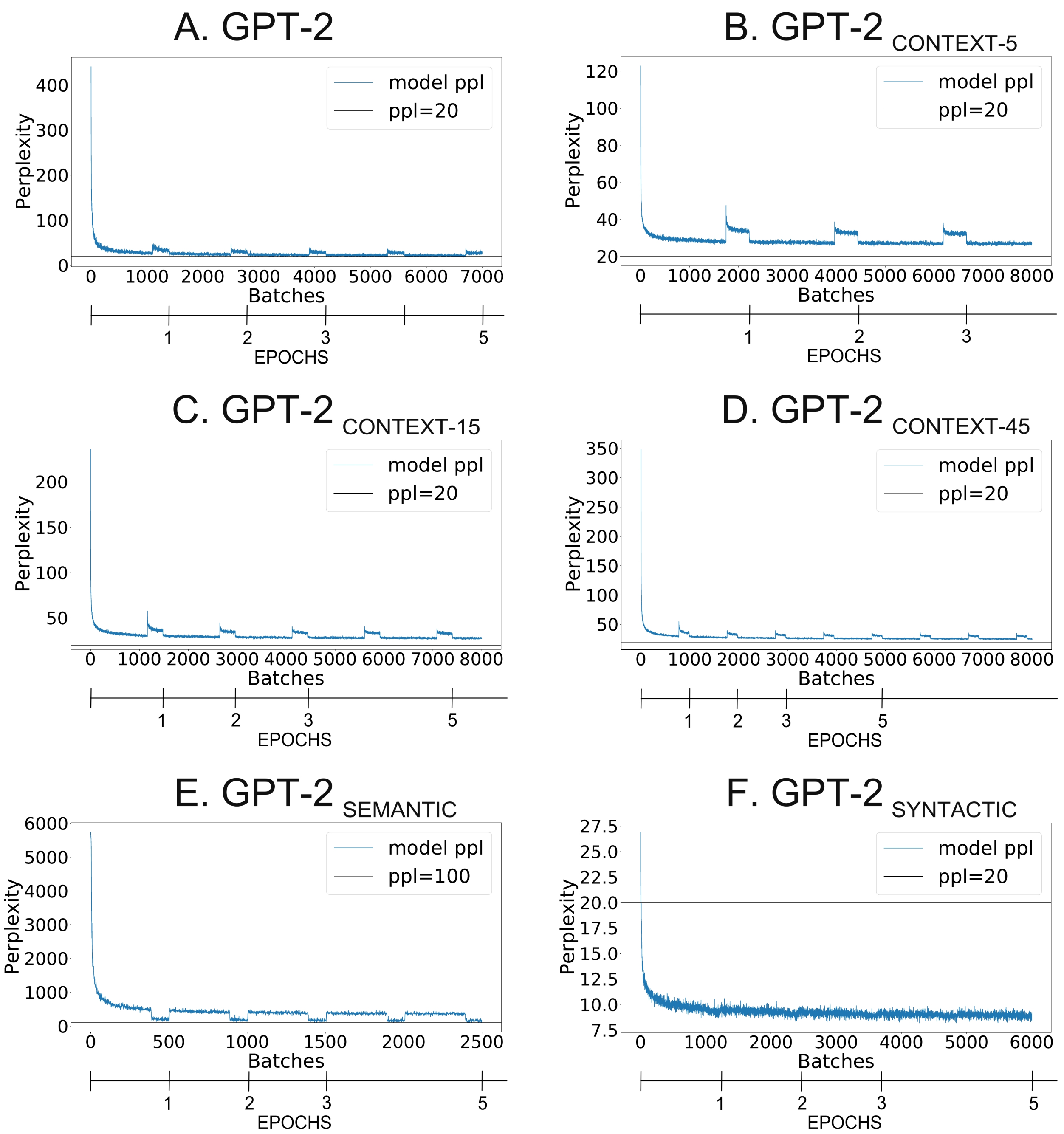


**Figure D1**

***GPT-2 convergence during training.*** *The models represented in panels A to D were trained on the integral features. Models in panels E and F were respectively trained on the semantic and syntactic features. Models were trained until no further improvement could be observed on the validation set.*

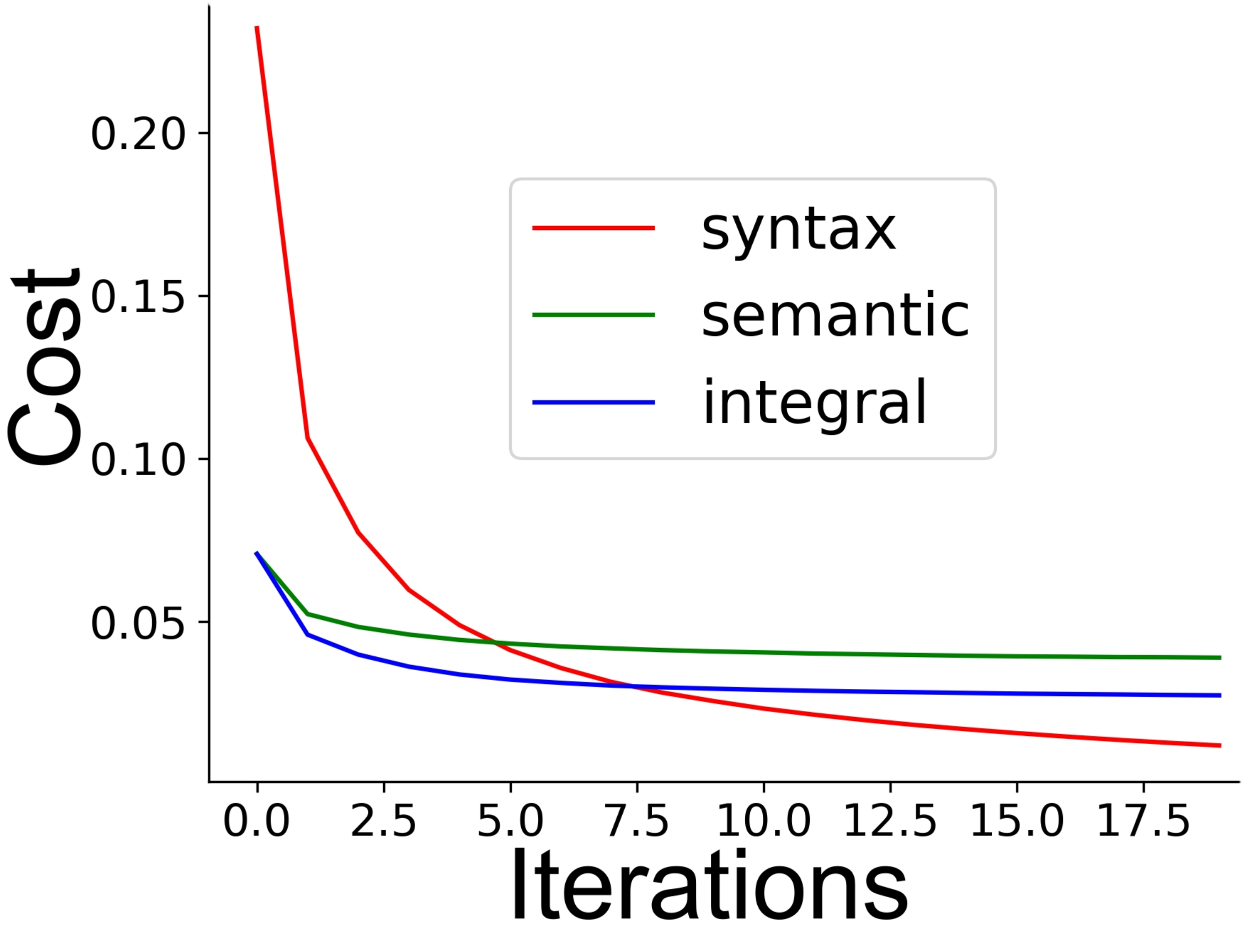


**Figure D2**

***GloVe convergence during training.*** *The models represented were trained on the integral features (blue), semantic features (green) or syntactic features (red). Models were trained until no further improvement could be observed on the validation set.*

## Appendix E

### Decoding individual syntactic features

Appendix 1-Fig.E1 shows the decoding accuracies of GloVe and GPT-2 models when trained on one of the three datasets. The syntactic labels varies from encompassing all categories (Morph+POS+NCN), to only one of them.

It is important to highlight that the decoding performance of the semantic models on the syntactic decoding task primarily relies on *Morph*. In contrast, the decoding of the Part-of-speech or the Number of Closing Nodes (NCN) are at chance level. This suggests that information related to the gender or plurals might be encoded by both syntactic and semantic embeddings. In addition, there are indeed more semantic labels compared to the syntactic ones. Consequently, the space occupied by syntactic embeddings is relatively smaller than that of semantic embeddings. As a result, it is relatively easier to project the larger semantic space onto the syntactic embedding space.

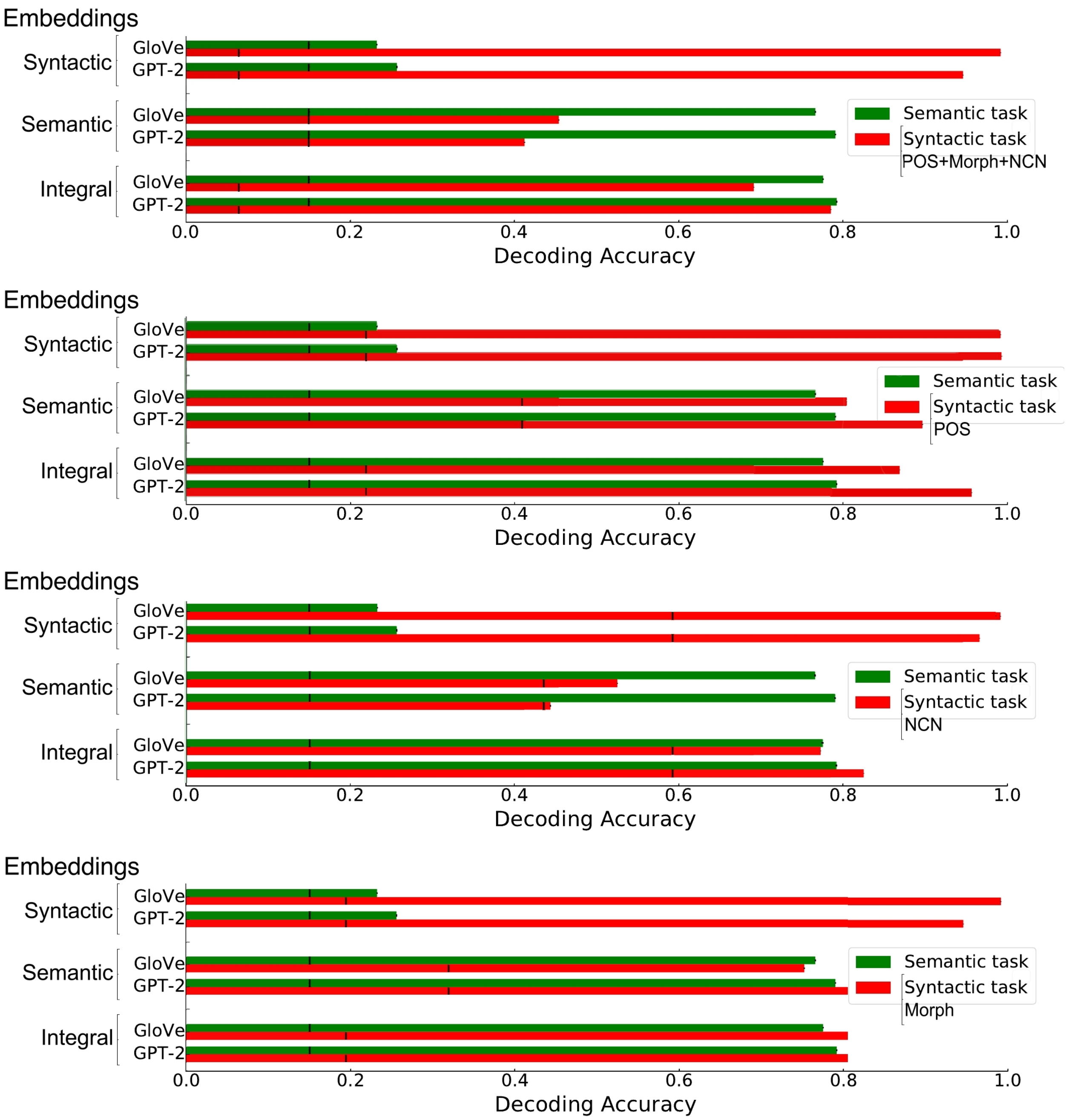


**Figure E1**

***Decoding syntactic information from words embeddings.*** *For each dataset and model type (Glove and GPT-2), logistic classifiers were set up to decode either the syntactic or the semantic categories of the words from the text of The Little Prince. Chance-level was assessed using dummy classifiers and is indicated by black vertical lines. From top to bottom: The syntactic label is i) the triplet (POS, Morph, NCN), ii) the POS, iii) the NCN, iv) the Morph.*

## Appendix F

## Mapping NLM activations to brain data

Given two non-linear transformations $\varphi_1$ (the neural language model that takes as input the sentence and from which we extract latent representations) and $\varphi_2$ (the brain that takes as input the sentence and from which we extract voxels' activations) and an input sequence $\mathbf{w} = (\mathrm{w}_1, \ . \ . \ . \ , \mathrm{w}_M)$, we define $\mathbf{Y}_s = \varphi_2(\mathbf{w}) \in \mathbb{R}^{N \times V}$ and $\mathbf{X} = \varphi_1(\mathbf{w}) \in \mathbb{R}^{M \times d}$, and we aimed at finding a linear transformation from $\mathbf{X}$ to $\mathbf{Y}_s$, where d is the dimension of the model, V is the number of brain voxels, and N the number of fMRI scans acquired. One issue is that $\mathbf{X}$ and $\mathbf{Y}_s$ don't have the same sampling frequency: $\mathbf{X}$ being defined at word-level while $\mathbf{Y}_s$ has been re-sampled at the fMRI acquisition frequency, every 2 seconds. To map $\mathbf{X}$ to $\mathbf{Y}_s$ we first need to temporally align them, taking the dynamic of the fMRI BOLD signal into account, and then determine a linear spatial mapping between the convolved and re-sampled $\mathbf{X}$ and $\mathbf{Y}_s$. Using the standard model-based encoding approach to modelling fMRI signals (Huth et al., 2016; Naselaris et al., 2011; Pasquiou et al., 2022), we first convolve each column of $\mathbf{X}$ with the *SPM* haemodynamic kernel (K), which corresponds to the profile of the fMRI BOLD response following a Dirac stimulation, and then sub-sampled the signal to match the sampling frequency of $\mathbf{Y}_s$, giving $\tilde{\mathbf{X}} = S_{ub}(K \circ X)$, with $S_{ub}$ the sub-sampling operator. Finally, we learn the linear spatial mapping between $\tilde{\mathbf{X}}$ and $\mathbf{Y}_s$ using a nested cross-validated L2-regularized (aka Ridge) univariate linear encoding model. More precisely, for each voxel $\mathbf{y}_s^v$, we learn a linear projection $\hat{\boldsymbol{\beta}}_s^v$ from $\tilde{\mathbf{X}}$ to $\mathbf{y}_s^v$ using a nested cross-validated L2-regularized univariate linear encoding model whose general solution is given by:

$$\hat{\boldsymbol{\beta}}_{\boldsymbol{s}}^{\boldsymbol{v}} = arg \min_{\boldsymbol{\beta_s}} \|\mathbf{y}_s^v - \boldsymbol{\beta}_{\boldsymbol{s}}^T \mathbf{X}\|^2 + \lambda \|\boldsymbol{\beta_s}\|_2^2 \text{ i.e. } \hat{\boldsymbol{\beta}}_s = \mathrm{Ridge}(\mathbf{X}, \boldsymbol{Y_s})$$

The latter stage resulted for each model and each run into a design matrix $\mathbf{X}$ of size $N \times d$. Given a neural language model, we gave the associated nine design-matrices to a nested cross-validated L2-regularized univariate linear encoding model to fit the fMRI brain data (of size $N \times V$). To evaluate model performance and the optimal regularization parameter

$\lambda^*$, we used a nested cross-validation procedure: we split each participant's dataset into training, validation and test sets, such that the training set included 7 out of the 9 experiment runs, and the validation and test sets contained one of the two remaining sessions. We evaluated model performance using Pearson correlation coefficient $R$, which is a measure of the linear correlation between encoding models' predicted time-courses and the actual time-courses. For each subject and each voxel, we first determined $\lambda^*$ by comparing $R_{valid}$ for 10 different values of $\lambda$, linearly spaced in log-scale between $10^{-3}$ and $10^4$. We then calculated $R_{test}$ for $\lambda^*$. Finally, we repeated this procedure 9 times, using cross-validation. This resulted in 9 $R_{test}$ values that we then averaged to produce a single $R_{test}$ map for the participant. We evaluated the quality of the mapping for subject $s$ in voxel $v$ using Pearson correlation:

$$R(X)_s^v = \mathrm{Corr}(\mathbf{Y_s^v}, \hat{\boldsymbol{\beta}}_\mathbf{s}^\mathbf{v}\mathbf{X})$$

## Appendix G

### The Basic Features baseline model

To assess the specific impact of NLMs' embeddings, the maps shown in Fig.3 report *increases in R values* relative to a *baseline model* which comprised three variables of non-interest:

- acoustic energy (root mean squared of the audio signal sampled every 10ms)
- word offsets (one event at each word offset)
- log of the lexical frequency of each word (modulator of the words events).

More generally, as we looked at increases in $R$ scores between models, the baseline model was appended to all other models studied in order to cancel out the effects of the 3 features of non-interest. Appendix 1-Fig.G1 below displays the cross-validated correlations obtained from this baseline model.

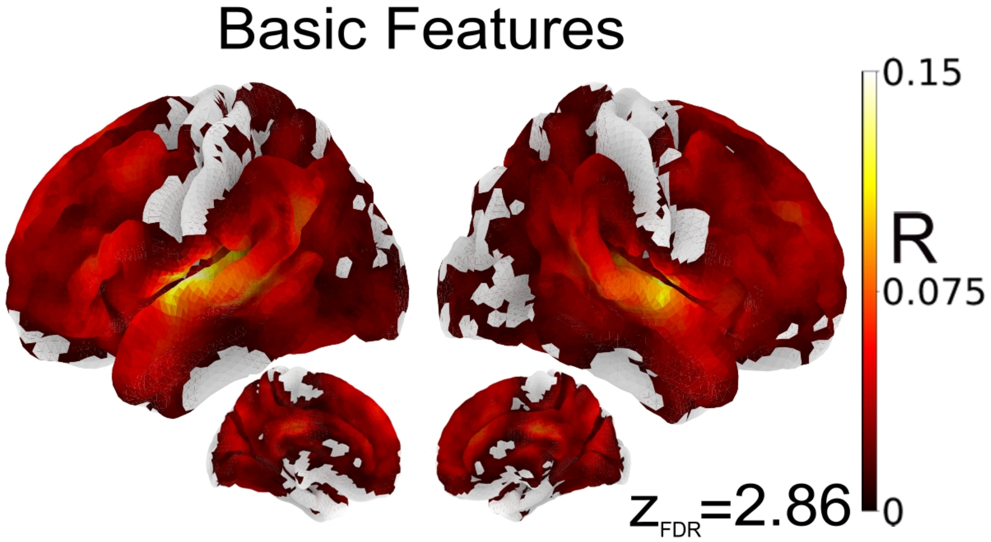


**Figure G1**
***Brain regions showing significant activations for the Basic Features baseline model.*** *Using the Basic Features (BF) baseline model to fit fMRI brain data, we displayed voxels where there was a significant correlation (voxel-wise thresholded group analyses; N=51 subjects; corrected for multiple comparisons with a FDR approach* $p < 0.005$*;* $z_{FDR}$ *is the FDR threshold on the z-scores). The effects from the Basic Features baseline model were discarded from all the analyses in the paper.*

## Appendix H

### Brain fit of GloVe and GPT-2 when trained on the Integral Features

Appendix 1-Fig.H1 shows the increase in $R$, relative to the baseline model, provided by the GloVe and GPT-2 models trained on the Integral Features, that is, the intact text.

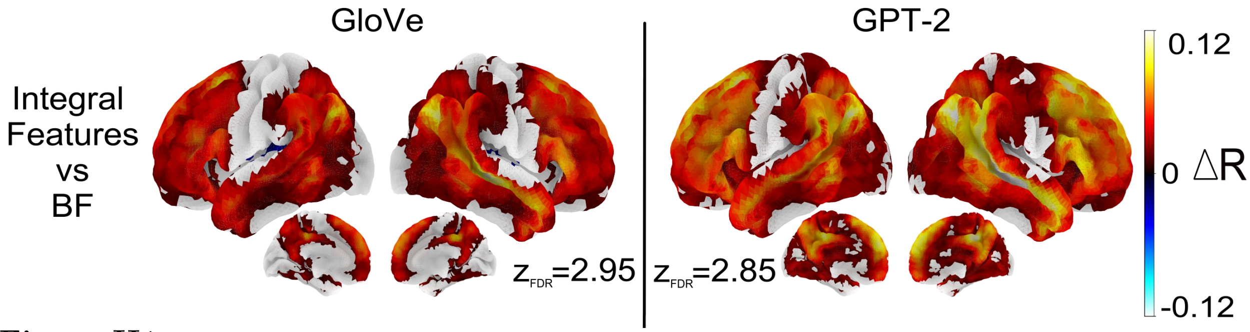


**Figure H1**

***Brain regions showing significant*** $R$ ***score increases compared to the Baseline Model for GloVe and GPT-2 when trained on the Integral Features.*** *Increases in R scores relative to the baseline model for GloVe (a non contextual model) and GPT-2 (a contextual model), trained on the Integral features (voxel-wise thresholded group analyses; N=51 subjects; corrected for multiple comparisons with a FDR approach* $p < 0.005$*;* $z_{FDR}$ *is the FDR threshold on the z-scores).*

# Appendix I

## R Scores Distribution for GloVe and GPT-2 Trained on Semantic or Syntactic Features

Appendix 1-Fig.I1 below shows the averaged (across participants) voxels distribution, of the increase in $R$ scores obtained from GloVe and GPT-2 models on semantic or syntactic features, relative to the baseline model.

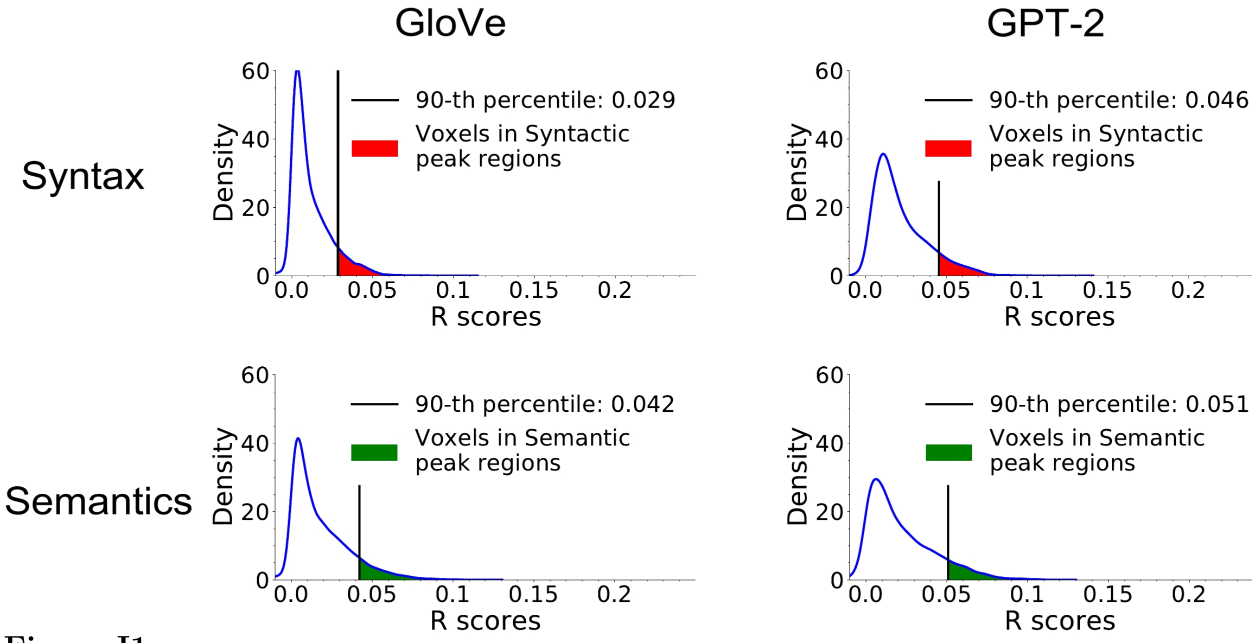


**Figure I1**

***Distribution of*** $R$ ***scores derived from GloVe and GPT-2 semantic and syntactic embeddings.*** *The 90th-percentile of the* $R$ *scores distribution is highlighted with a vertical black line and used to select voxels for the peak regions analyses.*

# Appendix J

## Models trained on Semantic features vs models trained on Syntactic features

Appendix 1-Fig.J1 shows the differences in R scores between the semantic and syntactic models, for Glove and GPT-2. Correcting for multiple comparisons (N=51; $p < 0.005$ after FDR correction), we observed significant differences in favor of the syntactic embeddings in the STG, and significant differences in favor of the semantic embeddings in the pMTG, the AG and the IFS and SFS.

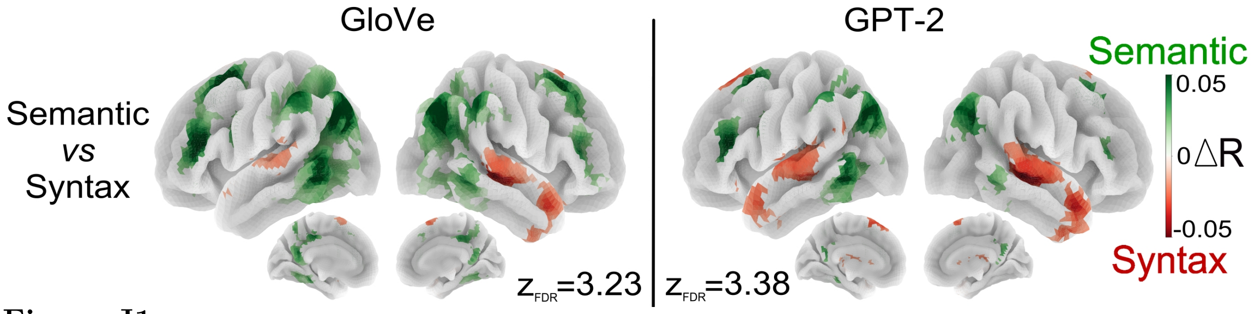


**Figure J1**

***Comparison of the models trained on Semantic features with the models trained on Syntactic features.*** *Significant R score differences between the models trained on Semantic features and the models trained on Syntactic features. The brain regions that are better fitted by the former model appear in green, while the regions better fitted by the latter model appear in red. (All these maps represent voxel-wise thresholded group analyses; N=51 subjects; corrected for multiple comparisons with a FDR approach* $p < 0.005$*).*

## Appendix K

### Semantic and syntactic peak regions at the subject-level

Appendix 1-Fig.K1 shows the peak regions analysis for GloVe and GPT-2 for the first 10 subjects. The figure shows syntactic peak regions around temporal regions and the dmPFC and semantic peak regions around the pMTG, AG, IFS and Precuneus. Appendix 1-Fig.K4 shows the distribution of Jaccard scores across subjects, separating the left hemisphere (in red) from the right (in blue).

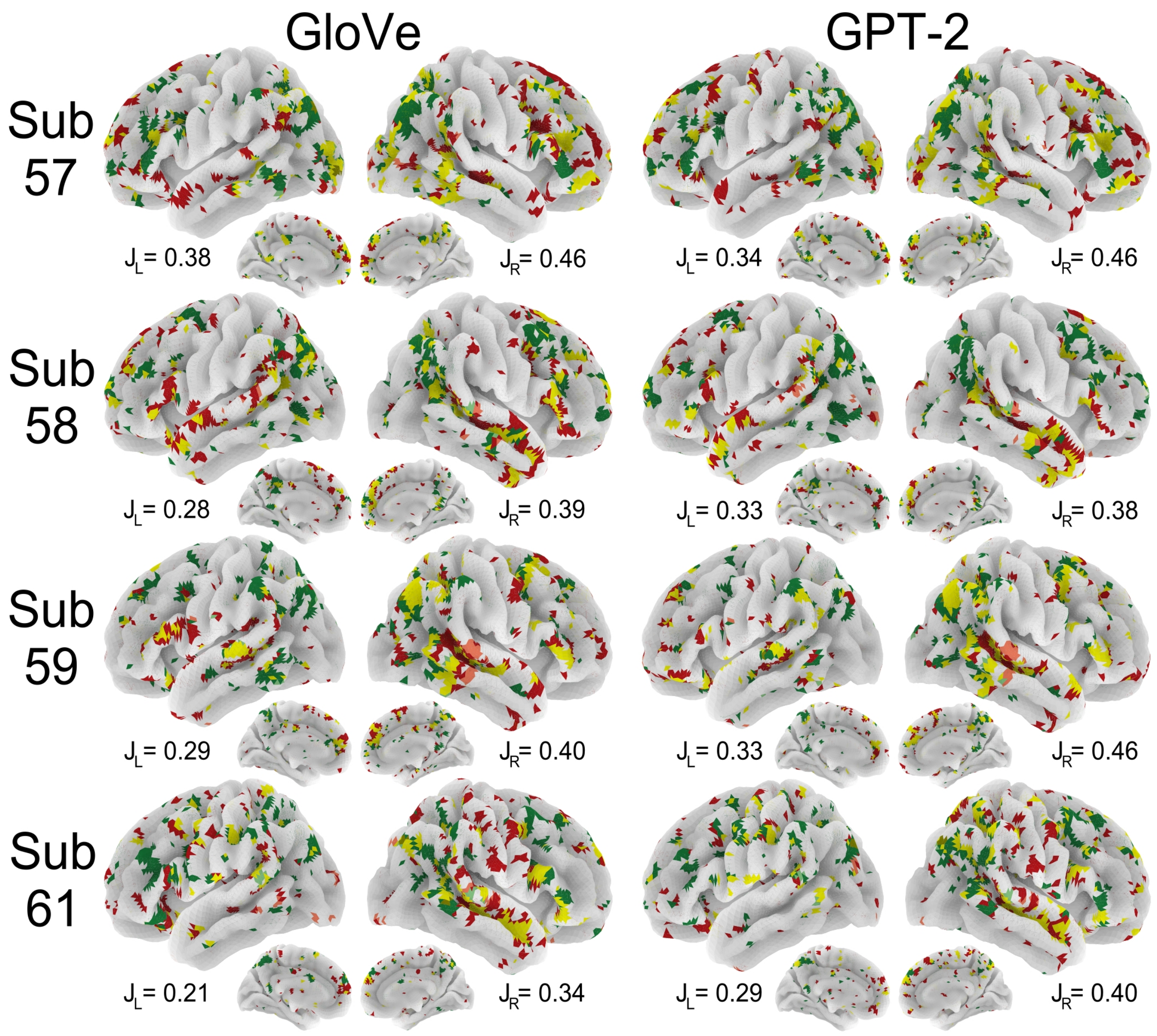

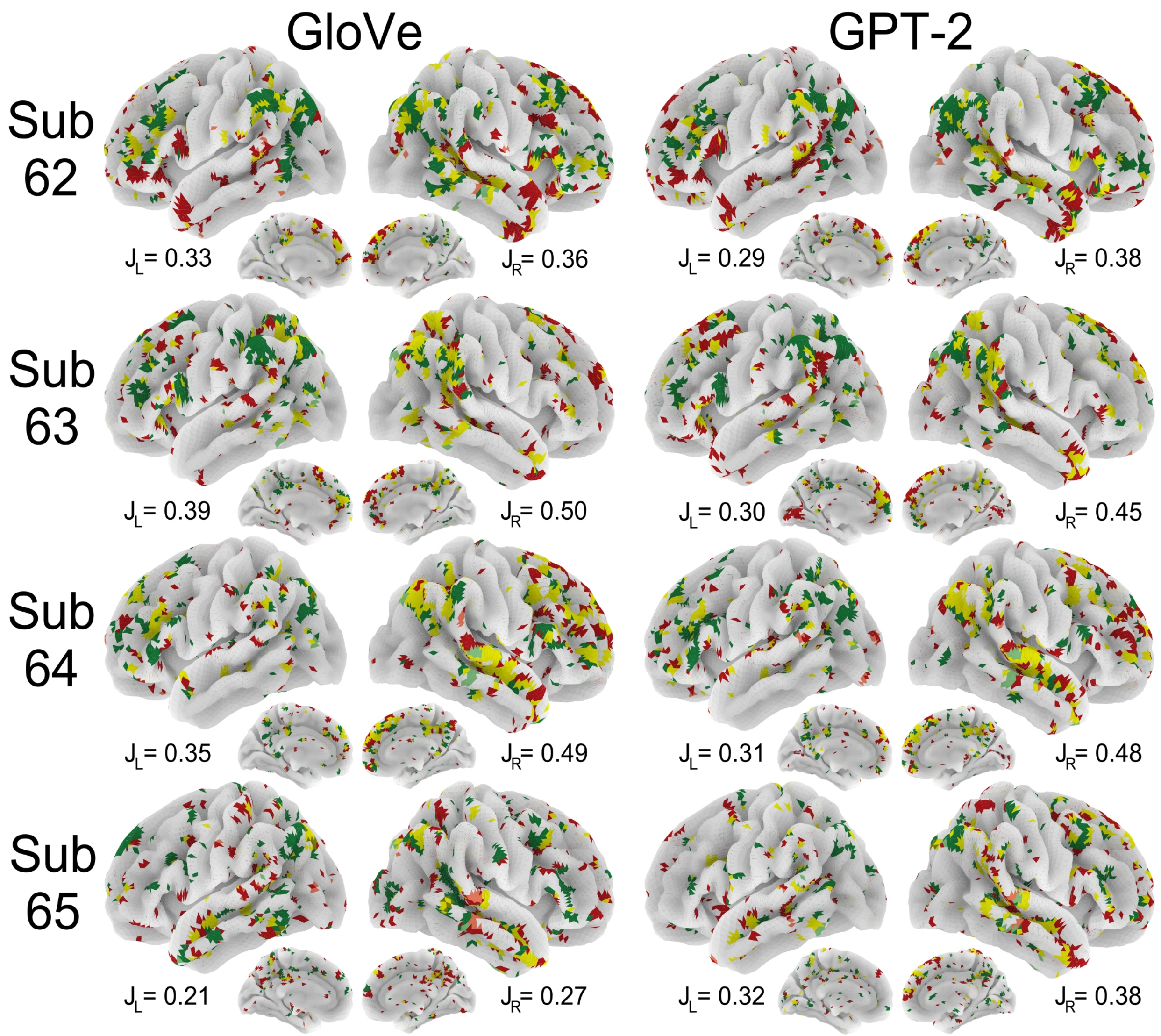


**Figure K1**

***Peak regions of syntax and semantics across subjects.*** *Bilateral spatial organisation of syntax and semantics highest R scores for the first 10 English subjects of The Little Prince fMRI corpus. Voxels whose R score belong in the 10% highest R scores (in green for models trained on the semantic features, and in red for models trained on the syntactic features) are projected onto brain surface maps for GloVe and GPT-2 (overlap in yellow and other voxels in grey). Jaccard score for each hemisphere are computed, i.e. the ratio between the size of the intersection and the size of the union of semantics and syntax peak regions.*

Appendix 1-Fig.K2 and Appendix 1-Fig.K3 show that the subject-level and group-level analyses are coherent with syntactic peak regions around the Temporal regions, the IFG and dmPFC, and semantic peak regions around the TPJ and the

Precuneous/posterior Cingulate gyri.

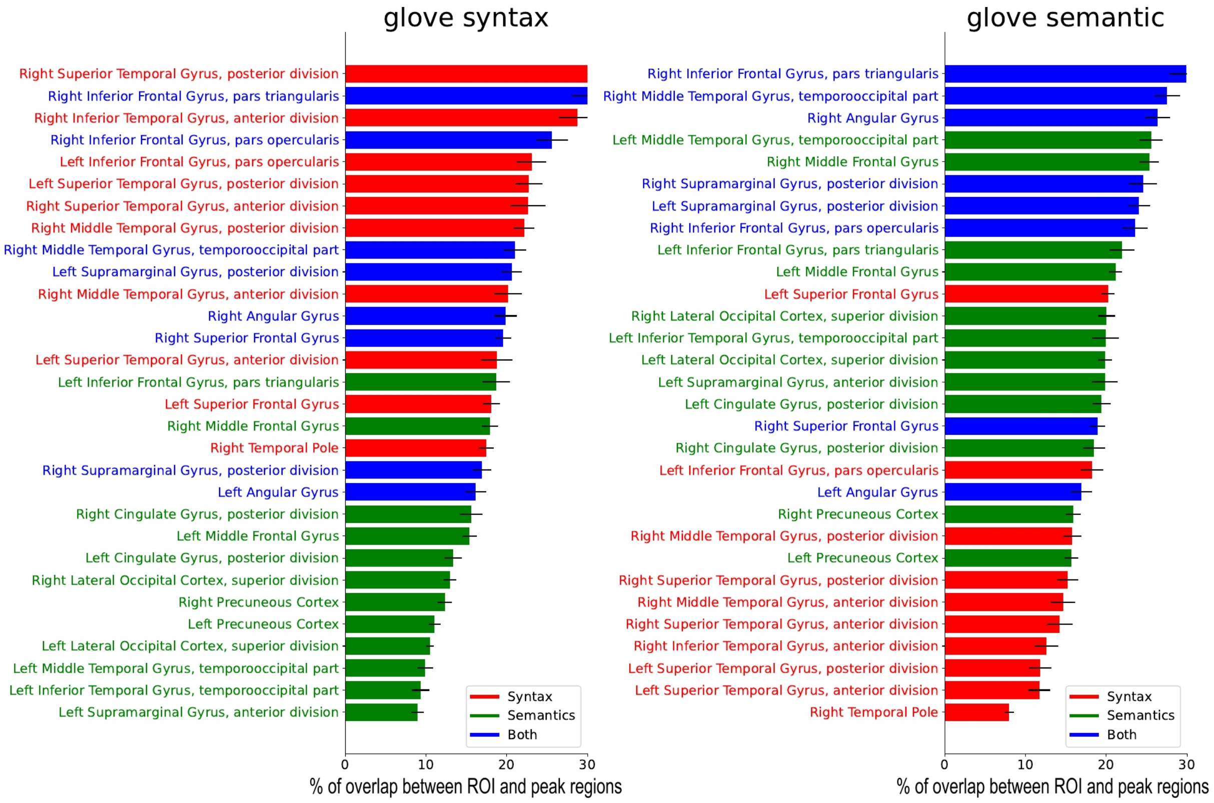


**Figure K2**

***Overlap between Harvard-Oxford ROIs and syntactic/semantic peak regions, averaged across subjects (for GloVe).*** *Percentage of voxels of the Harvard-Oxford ROIs that belong to the syntactic peak regions (left) and semantic peak regions (right), averaged across the 51 English subjects. The error bars display the standard error to the mean. Regions in red were identified as syntactic peak regions in the group-level analysis, while regions in green were identified as semantic peak regions. Regions in blue belong to both.*

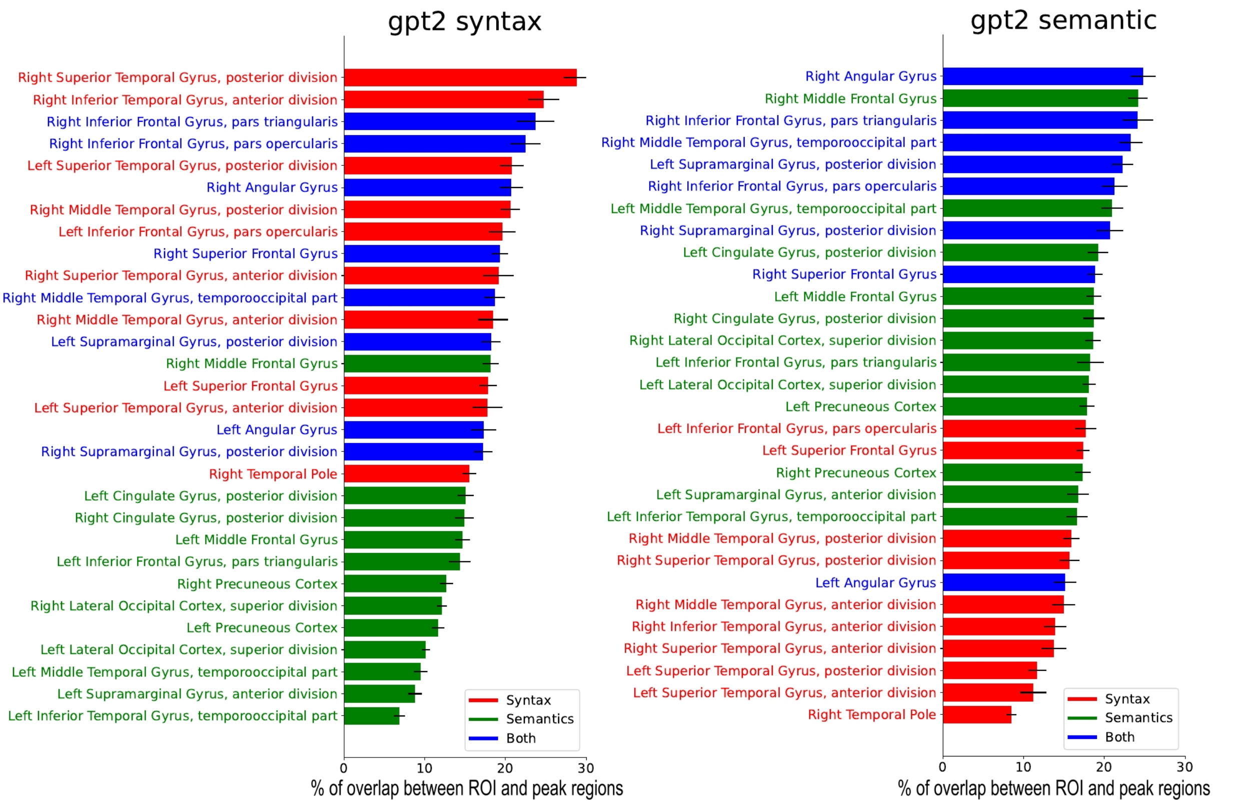


**Figure K3**

***Idem but for GPT-2***

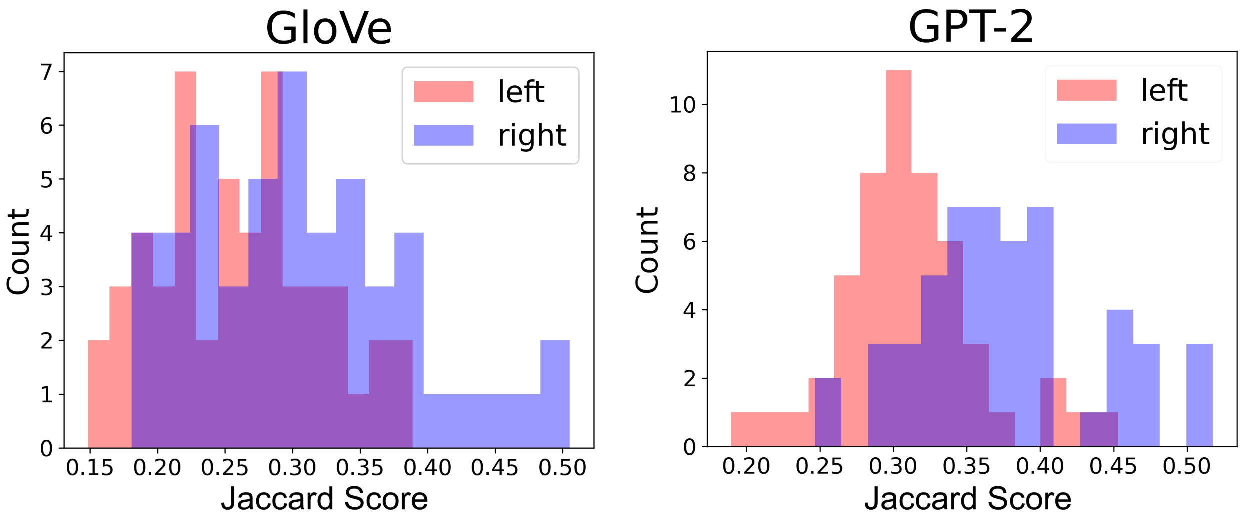


**Figure K4**

***Jaccard scores distribution across subjects.*** *Distribution of the Jaccard scores across the 51 English participants, in red for the left hemisphere and blue for the right.*

## Appendix L

### Layer-wise analysis

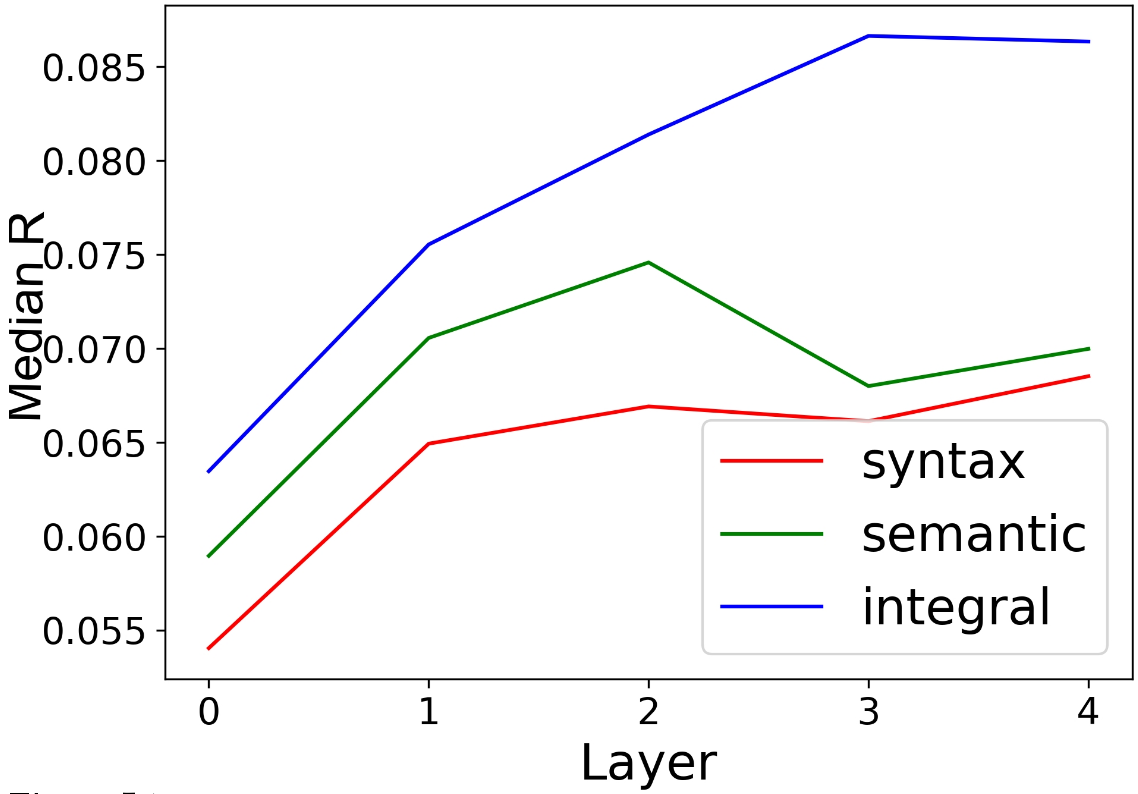


**Figure L1**

***Layer-wise analysis of the models trained on integral/semantic/syntactic features.*** *Impact of layer depth on the predictive power of GPT-2 when trained on the integral features (blue), the syntactic features (red) and the semantic features (green).*

We further demonstrate the relevance of using the late middle layers in the transformer models' architecture. We display the impact of layer depth on the, per-region, predictive power of BERT models[1] having different total number of layers.

[1] made available by GOOGLE at https://github.com/google-research/bert

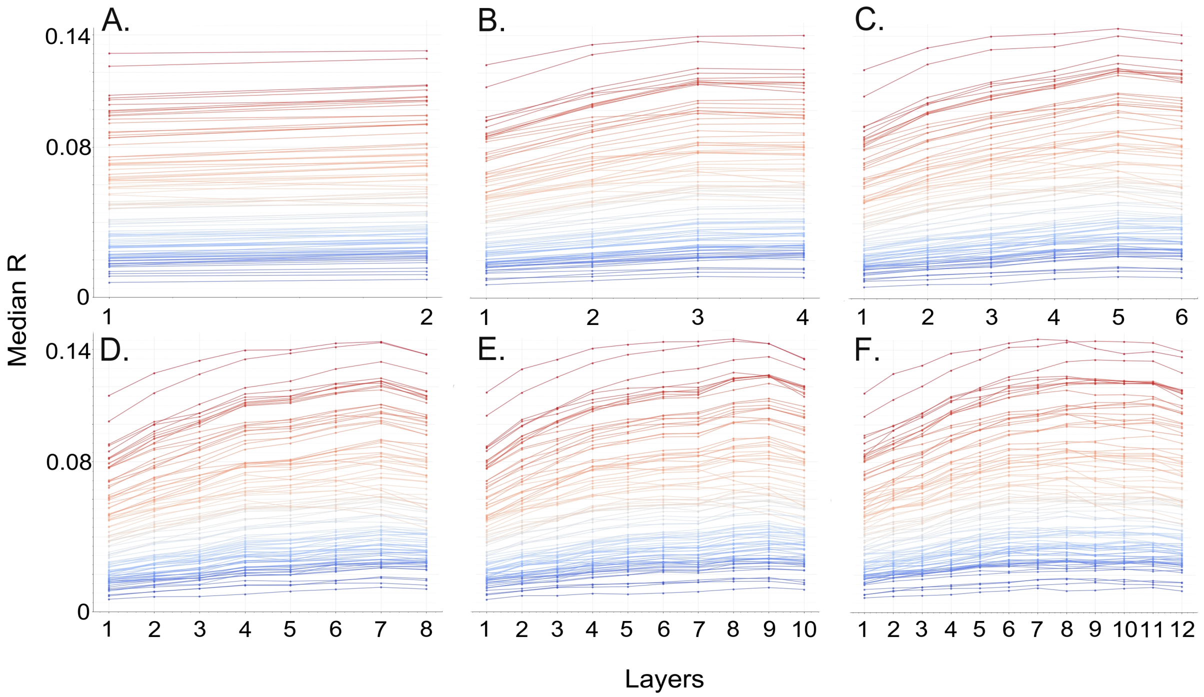


**Figure L2**

***Impact of layer depth on the, per-region, predictive power of BERT models having different total number of layers.*** *Impact of layer depth on the, per-region, predictive power of BERT models. A) 2-layer BERT, B) 4-layer BERT, C) 6-layer BERT, D) 8-layer BERT, E) 10-layer BERT, F) 12-layer BERT. Brain scores (median R values) were computed across voxels inside brain regions defined by the Harvard-Oxford atlas; each line corresponds to a region.*

# Appendix M

## Meta-Analysis based on Neurosynth

We used the *Neurosynth* database (https://github.com/neurosynth/neurosynth) to perform a meta-analysis of brain regions that appeared in fMRI articles containing the words 'syntactic' or 'semantic' in their abstract. Using a frequency threshold of 0.05, the keyword *semantic* yielded 626 articles, while *syntactic* yielded 128 articles.

The *meta.MetaAnalysis* function from the neurosynth package was then used to create association test maps for syntax and semantics. These maps display voxels that are reported more often in articles that mention the keyword than articles that do not. Such association test maps indicate whether or not there's a non-zero association between activation of the voxel in question and the use of a particular term in a study. We fused the maps associated to *syntactic* and *semantic*, thresholded with a False Discovery Rate set to 0.01, to produce Fig.M1.

In Fig.M1, we present the outcome of a meta analysis of the literature based on the search for the keywords 'syntactic' and 'semantic' in the Neurosynth database. This analysis, albeit somewhat simplistic, reveals the brain regions most often associated with syntax and semantics.

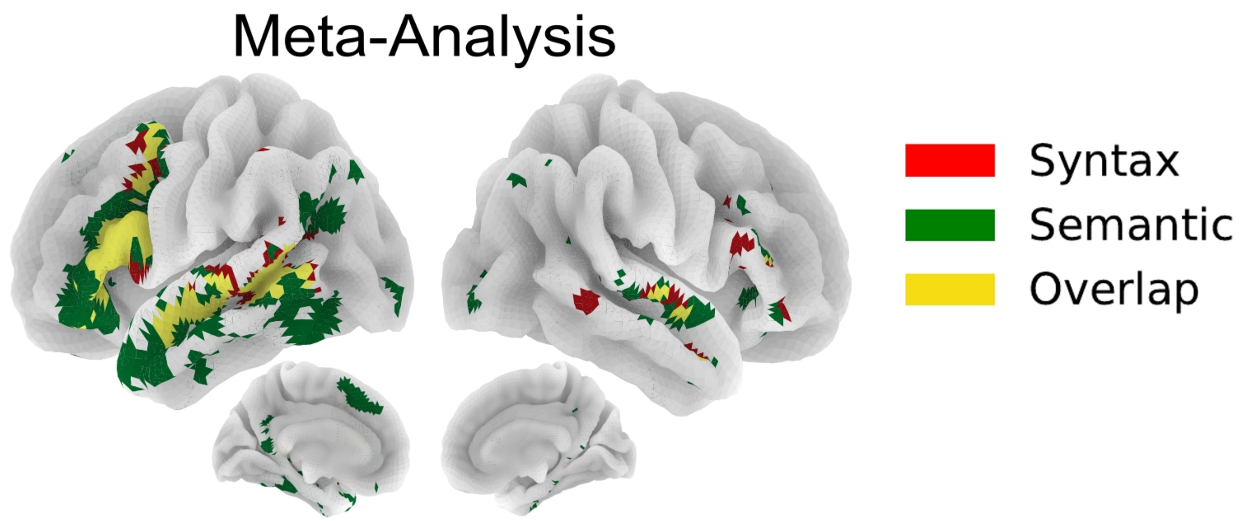


**Figure M1**

***Association maps for the terms "semantic" and "syntactic" in a meta-analysis using Neurosynth*** *(http://neurosynth.org) The association test map for syntactic (resp. semantic) displays voxels that are reported more often in articles that include the term syntactic (resp. semantic) in their abstracts than articles that do not (FDR correction of 0.01).*

## Appendix N

*

Brain Regions abbreviations

- STG: superior Temporal Gyrus
- STS: superior Temporal Sulcus
- TP: Temporal Pole
- IFG: inferior Frontal Gyrus
- IFS: inferior Frontal Sulcus
- DMPC: Dorso-Medial Prefrontal Cortex
- pMTG: posterior Middel Temporal Gyrus
- TPJ: temporo-parietal junction
- pCC: posterior Cingulate Cortex
- AG: Angular Gyrus
- SMA: Supplementary Motor Area
